# Aorta- and liver-generated TMAO enhances trained immunity for increased inflammation via ER stress/mitochondrial ROS/glycolysis pathways

**DOI:** 10.1172/jci.insight.158183

**Published:** 2023-01-10

**Authors:** Fatma Saaoud, Lu Liu, Keman Xu, Ramon Cueto, Ying Shao, Yifan Lu, Yu Sun, Nathaniel W. Snyder, Sheng Wu, Ling Yang, Yan Zhou, David L. Williams, Chuanfu Li, Laisel Martinez, Roberto I. Vazquez-Padron, Huaqing Zhao, Xiaohua Jiang, Hong Wang, Xiaofeng Yang

**Affiliations:** 1 Centers for Cardiovascular Research and; 2 Metabolic Disease Research and Thrombosis Research, Department of Cardiovascular Sciences, Lewis Katz School of Medicine at Temple University, Philadelphia, Pennsylvania, USA.; 3 Department of Medical Genetics and Molecular Biochemistry, Lewis Katz School of Medicine at Temple University, Philadelphia, Pennsylvania, USA.; 4 Biostatistics and Bioinformatics Facility, Fox Chase Cancer Center, Temple Health, Philadelphia, Pennsylvania, USA.; 5 Department of Surgery, Center of Excellence in Inflammation, Infectious Disease and Immunity, Quillen College of Medicine, East Tennessee State University, Johnson City, Tennessee, USA.; 6 DeWitt Daughtry Family Department of Surgery, Leonard M. Miller School of Medicine, University of Miami, Miami, Florida, USA.; 7 Center for Biostatistics and Epidemiology, Department of Biomedical Education and Data Science, Lewis Katz School of Medicine at Temple University, Philadelphia, Pennsylvania, USA

**Keywords:** Immunology, Inflammation, Cardiovascular disease, Chronic kidney disease, Innate immunity

## Abstract

We determined whether gut microbiota-produced trimethylamine (TMA) is oxidized into trimethylamine N-oxide (TMAO) in nonliver tissues and whether TMAO promotes inflammation via trained immunity (TI). We found that endoplasmic reticulum (ER) stress genes were coupregulated with MitoCarta genes in chronic kidney diseases (CKD); TMAO upregulated 190 genes in human aortic endothelial cells (HAECs); TMAO synthesis enzyme flavin-containing monooxygenase 3 (FMO3) was expressed in human and mouse aortas; TMAO transdifferentiated HAECs into innate immune cells; TMAO phosphorylated 12 kinases in cytosol via its receptor PERK and CREB, and integrated with PERK pathways; and PERK inhibitors suppressed TMAO-induced ICAM-1. TMAO upregulated 3 mitochondrial genes, downregulated inflammation inhibitor DARS2, and induced mitoROS, and mitoTEMPO inhibited TMAO-induced ICAM-1. β-Glucan priming, followed by TMAO restimulation, upregulated TNF-α by inducing metabolic reprogramming, and glycolysis inhibitor suppressed TMAO-induced ICAM-1. Our results have provided potentially novel insights regarding TMAO roles in inducing EC activation and innate immune transdifferentiation and inducing metabolic reprogramming and TI for enhanced vascular inflammation, and they have provided new therapeutic targets for treating cardiovascular diseases (CVD), CKD-promoted CVD, inflammation, transplantation, aging, and cancer.

## Graphical abstract

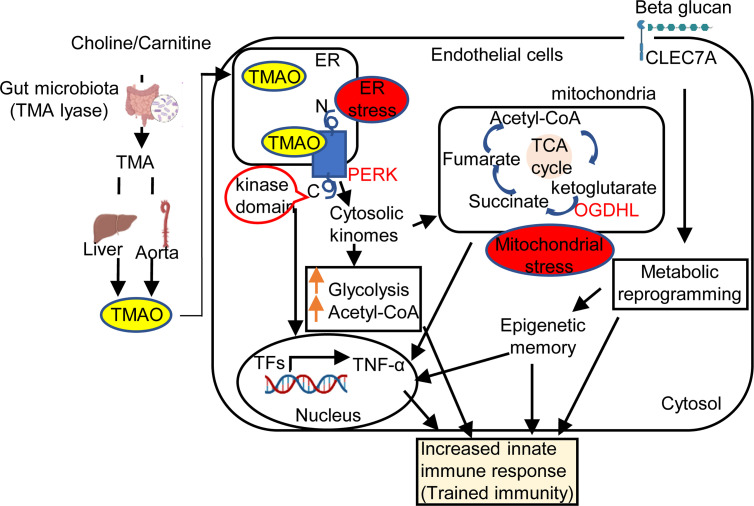

## Introduction

Vascular inflammation contributes significantly to the onset and complications of atherosclerosis (1–5). The Canakinumab Antiinflammatory Thrombosis Outcomes Study (CANTOS) demonstrated that the inhibition of proinflammatory IL-1β reduces the atherosclerotic burden in cardiovascular disease (CVD) (6). We proposed the following concepts: (a) endothelial cells (EC) are innate immune cells (3–5, 7–12); (b) activated ECs are characterized by upregulation of danger-associated molecular pattern (DAMP) receptors and major histocompatibility complex (MHC) molecules (13), in addition to upregulation of adhesion molecules and cytokines/chemokines; (c) endogenous metabolites that bind to their intrinsic receptors rather than classical DAMP receptors such as TLRs can become conditional DAMPs (14–17); and (d) ECs have innate immune memory functions (trained immunity [TI]) (2, 3, 18–20). However, how conditional DAMPs such as gut microbiota–generated uremic toxin (UT) (21, 22) trimethylamine N-oxide (TMAO) promote TI in human aortic ECs (HAECs) remains poorly characterized.

Conditional DAMPs, including lysophospholipids, have several key characteristics (9–12, 14–17, 23); they (a) are endogenous metabolites; (b) are elevated pathologically; (c) have physiological signaling roles; and (d) bind to their intrinsic receptors and carry out signal amplification (23). TMAO fits our conditional DAMPs category with the following aspects. (a) TMAO is derived from TMA by flavin-containing monooxygenase 3 (FMO3) oxidation mainly in the liver. TMA is generated by gut microbiota (TMA lyase) from choline, L-carnitine, betaine, and lecithin in the protein-rich diet and is reabsorbed into circulation via portal vein (22, 24). (b) TMAO binds to its receptor PERK (eukaryotic translation initiation factor 2 alpha kinase 3 [EIF2AK3]) (25) rather than commonly shared DAMP receptors. (c) TMAO is highly elevated in pathological conditions such as end-stage renal disease (ESRD) (26) and induced vascular inflammation in human umbilical vein ECs (HUVECs) (27). Finally, (d) TMAO initiates pathological signaling. TMAO promotes atherosclerosis in apolipoprotein E–KO (ApoE^–/–^) mice by inducing EC pyroptosis and activates NLRP3 inflammasome (27–31).

Innate immune cells, including ECs, can develop exacerbated immunologic response following brief exposure to endogenous/exogenous PAMPs/DAMPs, leading to an altered response and metabolic reprogramming toward a second challenge after the return to a nonactivated resting state. This phenomenon is known as TI (1–5, 18–20, 32). TI can be primed by a variety of stimuli, including β-glucan, LPS, Bacillus Calmette-Guerin (BCG), Western diet, and oxidized low-density lipoprotein (oxLDL) (3, 33–38). Reprogramming of cellular metabolism that takes place in the trained cells includes increased glycolysis, glutaminolysis, increased accumulation of tricarboxylic acid cycle (TCA) metabolites and acetyl-coenzyme A, increased mevalonate synthesis leading to epigenetic remodeling, and increased gene transcription and production of TNF-α (2). However, whether TMAO induces metabolic reprogramming and TI in ECs for enhanced inflammation remains unknown.

To address this question, we performed RNA-Seq analysis, single-cell RNA-Seq (scRNA-Seq) analysis, kinome analysis, and Seahorse glycolysis assays. Our data show that FMO3 is expressed in human and mouse aortas; TMAO significantly induces transcriptomic, kinomic, and metabolic reprogramming and mitochondrial reactive oxygen species (mitoROS) generation in HAECs (17, 39, 40), which induces HAEC activation and transdifferentiation into innate immune cells via upregulating cytokines/chemokines, secretoms, and clusters of differentiations (CDs) (8, 41, 42). Inhibition of endoplasmic reticulum (ER) stress mediator and PERK (TMAO receptor), inhibition of glycolysis, and inhibition of mitoROS suppress TMAO-induced HAECs activation. Our results have provided insights regarding TMAO’s roles in inducing EC activation and transdifferentiation, inducing metabolic reprogramming and TI for enhanced vascular inflammation, and inducing new therapeutic targets for treating chronic kidney disease–promoted (CKD-promoted) CVD, immune diseases, transplantation, aging, and cancers.

## Results

### Some ER stress genes were coupregulated with MitoCarta genes in UT serum–treated HCAECs, PBMCs from ESRD, and CKD renal specimens.

**Figure 1:**
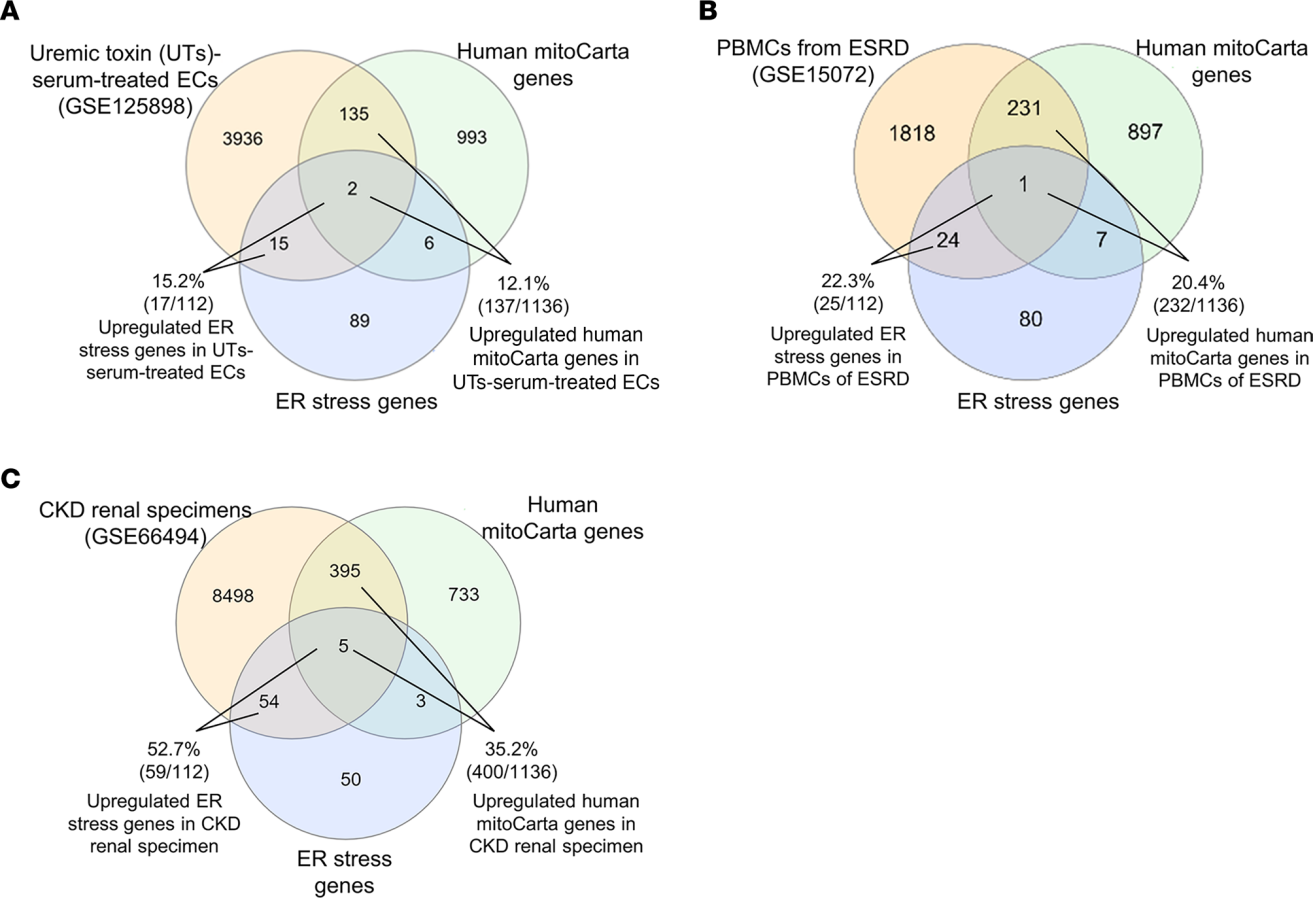
Some ER stress genes were coupregulated with MitoCarta genes in UT serum–treated HCAECs, PBMCs from ESRD patients, and CKD renal specimens. (**A**) CKD upregulated 15.2% of ER stress genes (PMID: 30027602, 18039139) and 12.1% of MitoCarta genes in UT serum–treated HCAECs. A complete gene list is shown in Supplemental Table 1A. (**B**) ESRD (GSE15072) upregulated 22.3% of ER stress genes and 20.4% of MitoCarta genes in PBMCs. A complete gene list is shown in Supplemental Table 1B. (**C**) CKD (GSE66494) upregulated 52.7% of ER stress genes and 35.2% of MitoCarta genes in CKD renal specimens. A complete gene list is shown the Supplemental Table 1C.

It has been reported that some UT induce ER stress in vascular smooth muscle cells (VSMC) (43) and human proximal tubular cells (44). However, an important question remains whether UT induce stresses in both ER and mitochondria. Of note, the upregulation of a partial list of nuclear genome–encoded mitochondrial genes (MitoCarta) were used as indicators of mitochondrial stress (45). We hypothesized that UT induce upregulation of ER stress genes and mitochondrial genes. To examine this hypothesis, we found 112 ER stress genes (46, 47) and 1,136 MitoCarta genes (48) to match with the significantly upregulated genes (fold change [FC] > 1.5 and *P* < 0.05) from RNA-Seq data set of UT serum–treated human coronary arterial endothelial cells (HCAECs) (GSE125898) (21, 49). In UT serum–upregulated genes, 17 (15.2%) ER stress genes were coupregulated with 137 (12.1%) MitoCarta genes (Figure 1A and Supplemental Table 1A; supplemental material available online with this article; https://doi.org/10.1172/jci.insight.158183DS1). We reasoned that, if this ER-mitochondrial–linked stress in ECs resulted from UT effects, then these findings should be observed in other cell types that were exposed to UT. We verified these findings in the significantly upregulated genes from transcriptomic data sets of peripheral blood mononuclear cells (PBMCs) from ESRD (GSE15072) (8), and microarray data sets from CKD renal specimens (GSE66494) (49, 50). In PBMCs of ESRD upregulated genes, 25 (22.3%) ER stress genes were coupregulated with 232 (20.4%) MitoCarta genes (Figure 1B and Supplemental Table 1B). In CKD renal specimen upregulated genes, 59 (52.7%) ER stress genes were coupregulated with 400 (35.2%) MitoCarta genes (Figure 1C and Supplemental Table 1C). Taken together, these results have demonstrated that parts of ER stress genes are coupregulated with parts of MitoCarta genes in UT serum–treated HCAECs, PBMCs of ESRD, and CKD renal specimens, suggesting a possibility that UT-induced ER stress may trigger mitochondrial stress in CKD; UT circulating in the blood of CKD/ESRD patients were shared in all 3 transcriptomic experimental settings.

### TMAO significantly reshaped transcriptome and upregulated 190 genes in HAECs.

**Figure 2:**
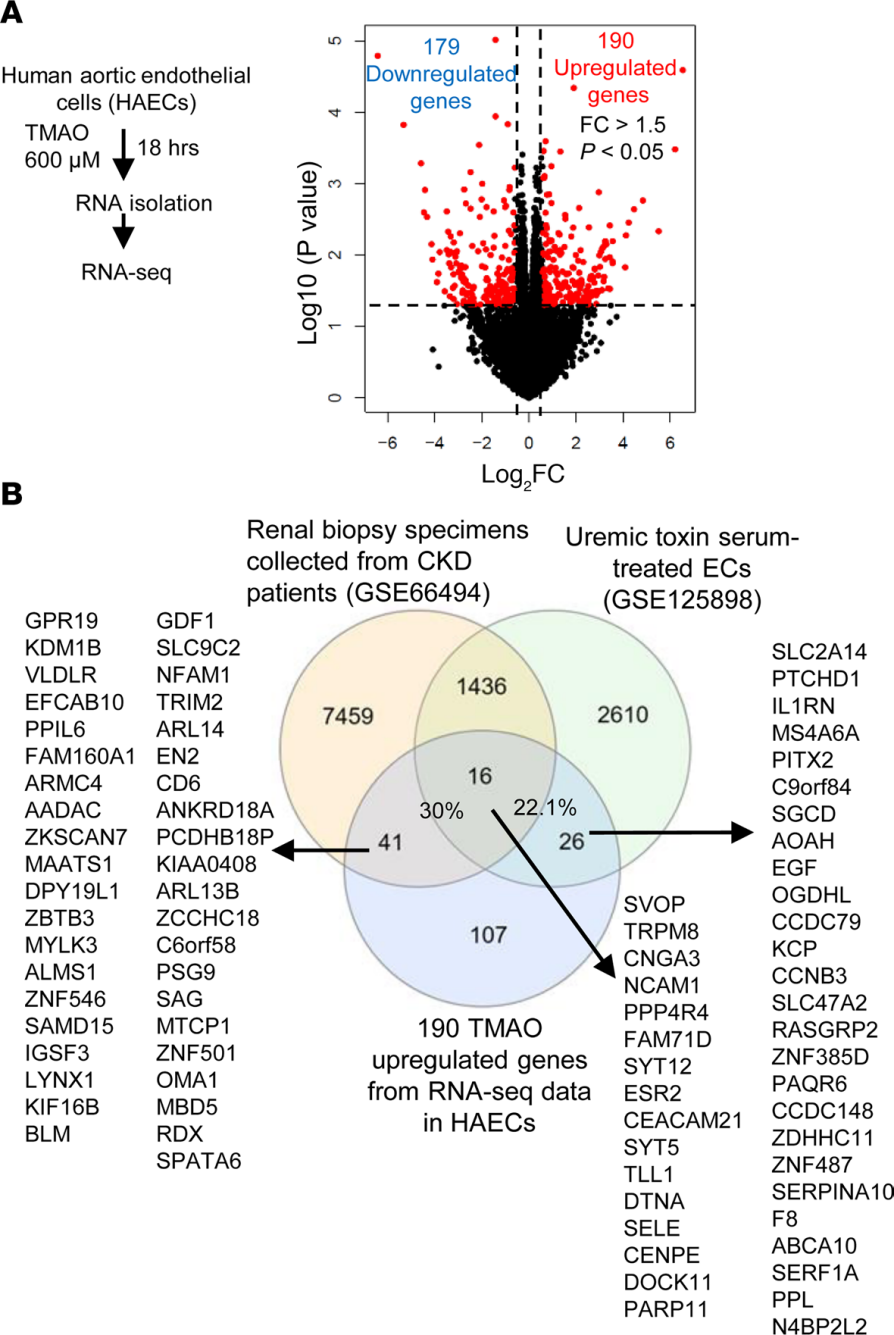
TMAO significantly reshaped transcriptome and upregulated 190 genes in human aortic endothelial cells (HAECs). (**A**) HAECs were treated with TMAO (600 μM) for 18 hours, and RNAs were collected for RNA-Seq (*n* = 3). The volcano plots showed the differentially expressed genes with *P* < 0.05 and FC > 1.5. TMAO significantly upregulated 190 and downregulated 179 genes. (**B**) In total, 30% of TMAO-upregulated genes were upregulated in CKD renal specimens and 22.1% of TMAO-upregulated genes were upregulated in UT serum–treated HCAECs.

To determine the molecular mechanisms underlying UT-induced ER stress–linked mitochondrial stress in human ECs and CKD accelerated vascular inflammation, we adopted the gut microbiota generated UT TMAO–treated HAECs as a UT stimulation model. One of the reasons to focus our study on TMAO was that a key ER stress mediator PERK was reported to be the receptor for TMAO (25, 51–53), which may provide a mechanistic link between ER stress and subsequent stresses in other intracellular organelles (54). We hypothesized that TMAO activates HAECs by modulating the transcriptome of HAECs. In our mechanistic study, we used TMAO of 600 μM (Figure 2A) because this concentration significantly increased expression of ICAM-1 and IL-1β, activated NLRP3 inflammasomes, and induced inflammation in HUVECs (26, 27, 55). RNA-Seq data show that TMAO significantly modulated the expression of 369 genes, with 190 genes upregulated and 179 genes downregulated (FC > 1.5, *P* < 0.05). Among 190 upregulated genes, 57 genes (30%) were shared with CKD renal upregulated genes, and 42 genes (22.1%) were shared with UT serum upregulated genes (Figure 2B). These results have demonstrated that TMAO-upregulated genes contribute significantly to UT-induced EC transcriptomic remodeling and CKD/UT-induced transcriptomic remodeling in renal tissues.

### TMAO-generating enzyme FMO3 was expressed in human and mouse aortic cells.

**Figure 3:**
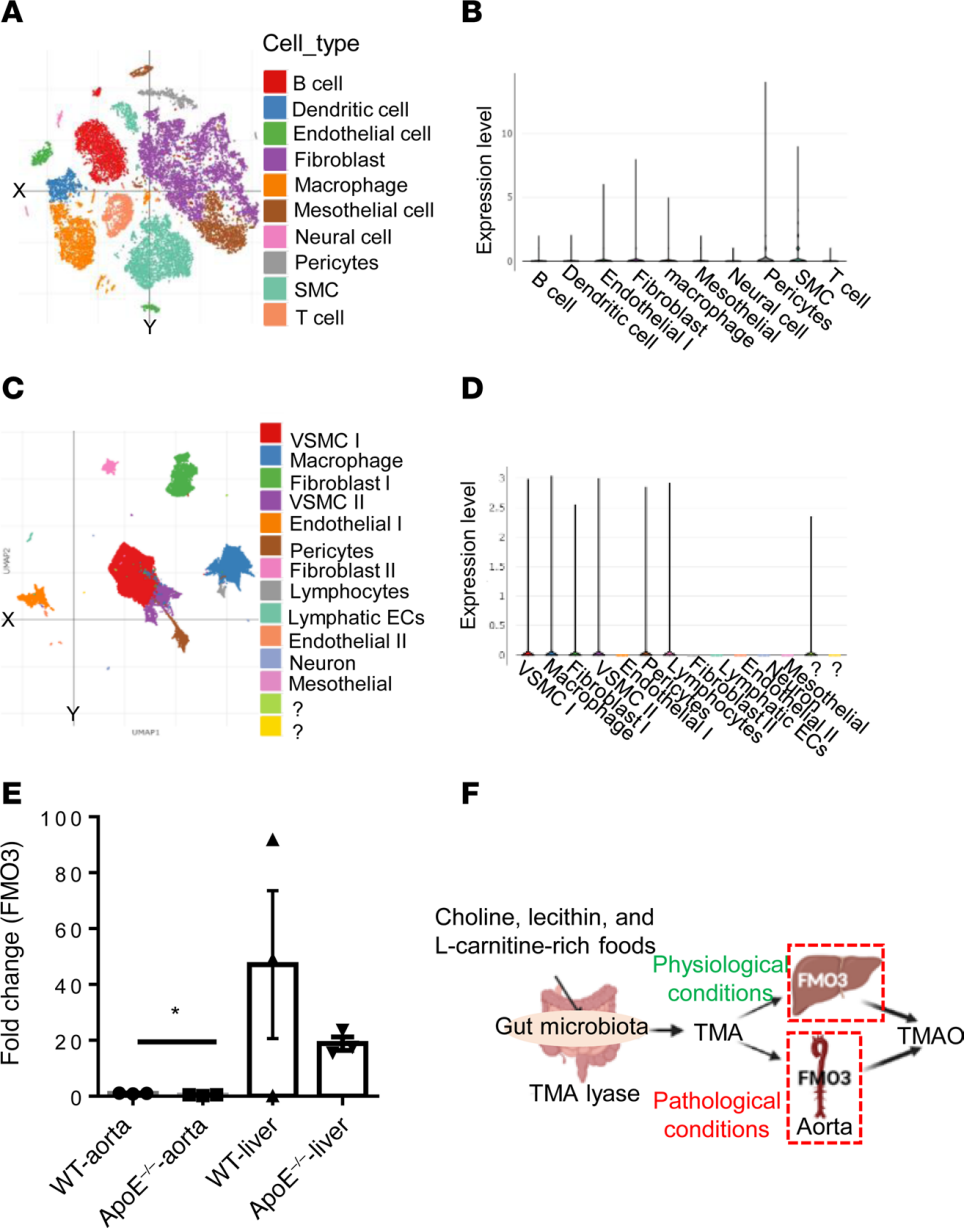
Extrahepatic expression of FMO3. scRNA-Seq data show that flavin-containing dimethylaniline monooxygenase 3 (FMO3) was expressed in the human aortic cells and aorta cells from mice fed with HFD, and aorta of WT and ApoE^–/–^ mice. (**A**) Single-cell transcriptome analysis of the ascending aortas of HFD-fed mice identified 10 cell types including ECs, fibroblasts, SMCs, B cells, T cells, macrophages, DCs, mesothelial cells, pericytes, and neural cells. (**B**) FMO3 expression in the aortic cells of HFD-fed mice. (**C**) Single-cell transcriptome analysis showed 12 cell types identified in the human thoracic aorta. (**D**) FMO3 expression in the human aortic cells. The data mining analyses were performed on the scRNA-Seq database of the Broad Institute of MIT and Harvard. (**E**) Real-time PCR showed the FMO3 expression in the aorta and liver of WT and ApoE^–/–^ mice (*n* = 3 samples in each group and each sample containing aortas and liver from 2 mice). (**F**) Schematic diagram showing the TMAO biogenesis under physiological conditions in liver tissue; however, the pathological conditions transform the aorta into TMAO-generating tissue (*t* test; *P < 0.05).

Kidney filtration dysfunction in CKD and ESRD patients results in increased generation and accumulation of UT (21). Thus, we hypothesized that FMO3 is upregulated in the aorta in CVDs. FMO3 is mainly expressed in human liver among 27 normal tissues (56). We examined the scRNA-Seq database at MIT-Broad Institute (Cambridge, Massachusetts, USA) (3). Among 10 cell types identified in the aortas of high-fat diet–fed (HFD-fed) mice, FMO3 was expressed in aortic ECs, fibroblasts, macrophages, pericytes, and SMCs (57) (Figure 3, A and B). In addition, other FMO family enzymes (58) were also expressed in the aortic cells of HFD-fed mice (Supplemental Figure 1, A–C). Furthermore, FMO3 expression was confirmed in different cell types in the human thoracic aorta, including VSMC I, VSMC II, fibroblasts, macrophages, pericytes, and lymphocytes (59) (Figure 3, C and D).

FMO3 expression has been reported in the aorta of C57BL/6 mice (60), human skin fibroblast, and human microvascular ECs (61). Therefore, we used quantitative PCR (qPCR) to measure the FMO3 expression in the aorta and liver of WT and ApoE^–/–^ mice fed with normal chow diet. We found that FMO3 was expressed in the liver and aortas of WT and ApoE^–/–^ mice (Figure 3E). Of note, in the normal chow diet–fed mice, FMO3 expression was higher in the aorta of WT than that of ApoE^–/–^ mice; however, these changes were statistically nonsignificant in the liver. Furthermore, other organs such as normal human heart (62), COVID-19 heart and lung (63), immune cell from human lung tumor (64), mouse glomerulus diseases (65), and aging mouse brain (66) can express FMO3 in different cell types (Supplemental Table 2) and can also express other FMOs family members (Supplemental Table 3). FMO3 deficiency confers protection against TMAO-induced obesity and modulation of energy metabolism (67). Taken together, since CKD promotes atherosclerosis (68) and aortic aneurysm (69), these results have demonstrated that different pathological conditions such as hyperlipidemia converts mouse and human aortas into TMAO-generating extrahepatic tissues (Figure 3F).

### TMAO activated and transdifferentiated HAECs into innate immune cells while upregulating 24 EC activation genes and adhesion molecules; 40 cytokines, chemokines, and secretoms; and 8 CDs.

**Figure 4:**
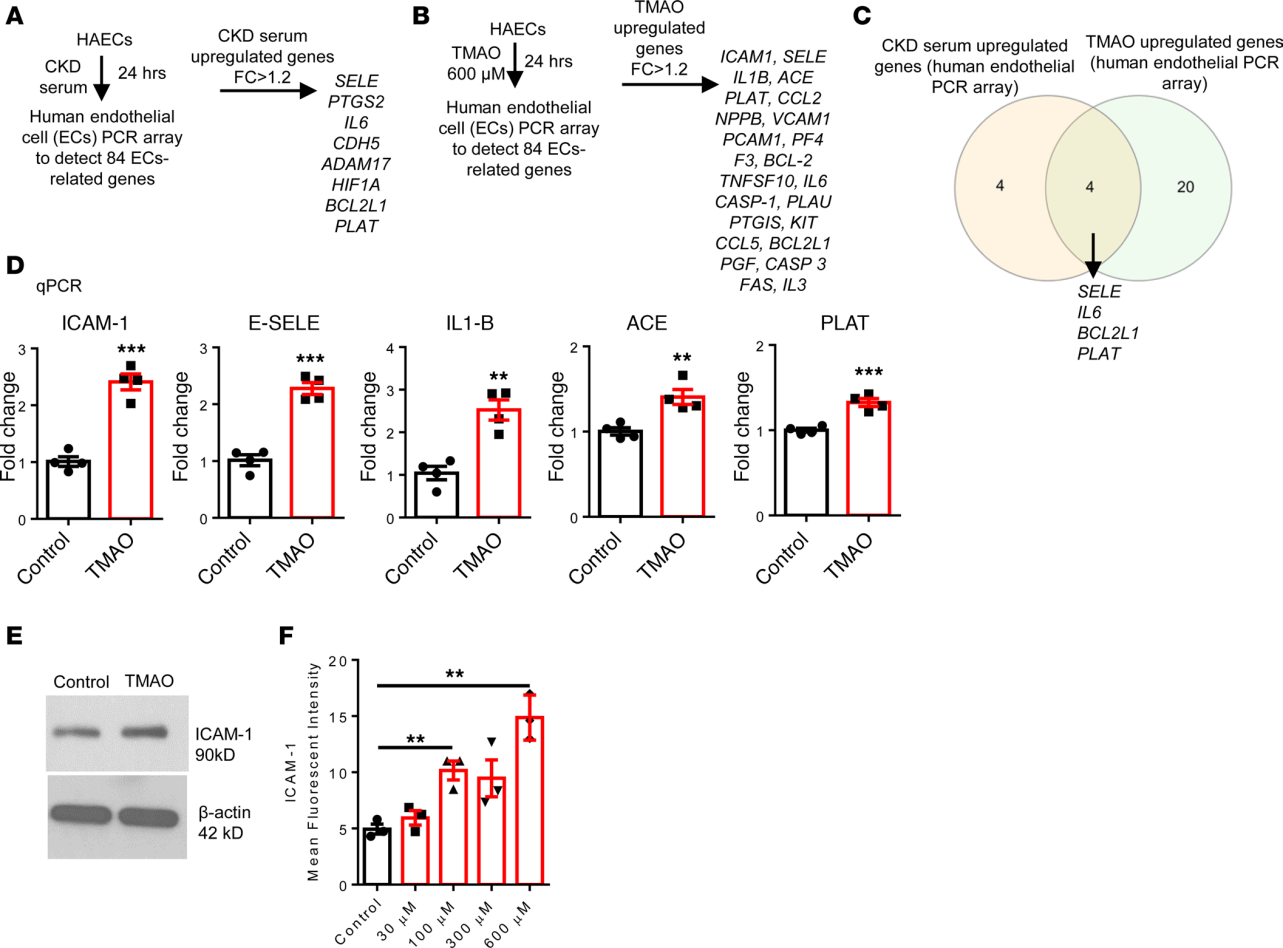
TMAO induces HAEC activation. HAECs were treated with pooled serum from 3 healthy individuals or 3 patients with CKD and TMAO (600 μM) for 24 hours. A human EC PCR array was used to detect 84 EC genes. (**A**) CKD serum upregulated 8 genes. (**B**) TMAO upregulated 24 genes. (**C**) Four EC genes overlapped between CKD serum– and TMAO-upregulated genes. (**D**) Real-time PCR analysis to verify some of the TMAO-upregulated genes (*n* = 4). (**E**) Western blot showed ICAM-1 expression. (**F**) Flow cytometry analysis shows that TMAO upregulated ICAM-1 expression (*n* = 3; each experiment was repeated 3 times). Data are represented as the mean ± SEM (*t* test; **P < 0.01, ***P < 0.001).

To determine the roles of TMAO in HAEC activation, we used a more focused EC PCR array. Eight EC-related genes were upregulated in UT serum–treated HAECs (Figure 4A), and 24 genes were upregulated in TMAO-treated HAECs (Figure 4B). Four of 8 UT serum–upregulated genes were shared with the TMAO-upregulated genes, including *SELE*, *IL-6*, *BCL2L1*, and *PLAT* (Figure 4C). From the TMAO-upregulated genes, 5 selected genes, including *ICAM-1*, *IL-1β*, *SELE*, *ACE*, and *PLAT*, from 24 were verified by qPCR (Figure 4D). Western blot (Figure 4E) and FACS analysis (Figure 4F) were further used to confirm the ICAM-1 protein upregulation by TMAO.

**Figure 5:**
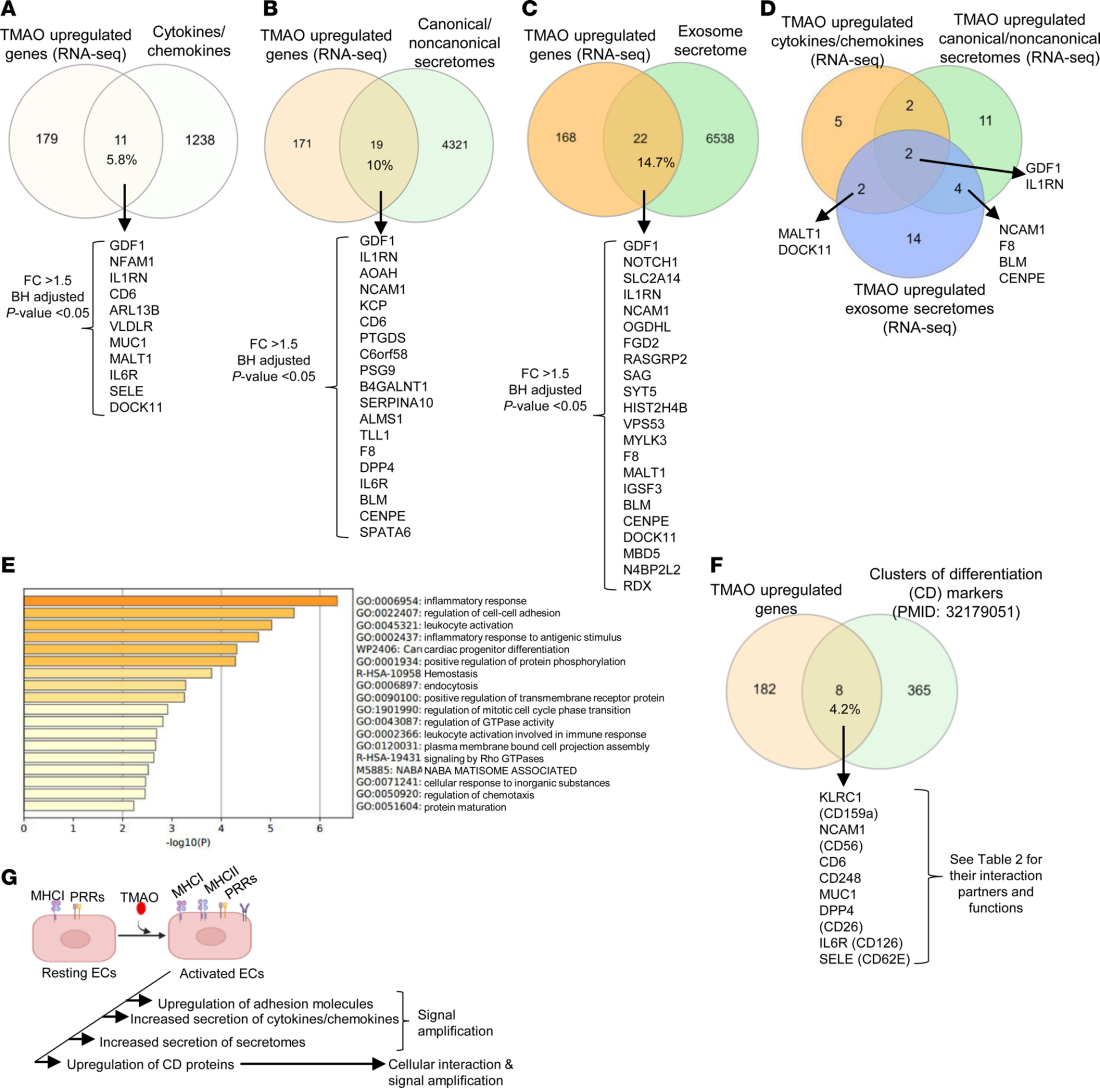
TMAO activated and transdifferentiated HAECs into innate immune cells by upregulating 24 EC genes and adhesion molecules; 40 cytokines, chemokines, and secretome genes; and 8 CDs. (**A**–**C**) RNA-Seq data show that TMAO significantly upregulated 11 cytokines/chemokines, 19 canonical/noncanonical secretomes, and 22 exosome secretomes. (**D**) The overlap between TMAO-upregulated cytokines/chemokines, canonical/noncanonical secretomes, and exosome secretomes. (**E**) Metascape pathway analysis showed the top pathways of the TMAO-upregulated 11 cytokines/chemokines, 19 canonical/noncanonical secretomes, and 22 exosome secretomes. (**F**) TMAO upregulated 8 CDs. (**G**) Schematic diagram showing resting and activated/transdifferentiated ECs by upregulating adhesion molecules, cytokines/chemokines, secretomes, and CDs to increase cellular interaction and signal amplification. The Benjamini-Hochberg Procedure (BH) was used to calculate the adjusted *P* value.

ECs are innate immune cells in CVDs (2–5). Our previous reports show that conditional DAMPs, such as lysophosphatidylcholine (lysoPC) and lysophosphatidylinositol (lysoPI), transdifferentiate HAECs into functional innate immune cells by upregulating adhesion molecules, cytokines/chemokines, and MHC molecules (13, 70). We hypothesized that TMAO promotes innate immune transdifferentiation of HAECs. We used a potentially novel knowledge-based transcriptomic profiling approach and Venn diagram analysis of TMAO-upregulated genes with 1,249 Ingenuity Pathway Analysis–designed (IPA-designed) cytokine/chemokine genes; 4,340 canonical secretomic genes; and noncanonical secretomic genes including 964 caspase-1–gasdermin D secretomes; 1,223 caspase-4 (humans)/-11 (mice) secretomes; and 6,560 exosome secretomic genes (70–73). We found that 11 cytokines/chemokines (Figure 5A), 19 canonical/noncanonical secretomic genes (Figure 5B), and 22 exosome secretomic genes (Figure 5C) were overlapped with the TMAO-upregulated genes (RNA-Seq). The 3 groups of TMAO-upregulated cytokines/chemokines and secretomic genes were partially overlapped (Figure 5D). Then, we used the Metascape pathway analyses (74) to determine the top functional pathways of TMAO-upregulated cytokines/chemokines and secretomic genes. The top signaling pathways include inflammatory response, regulation of cell-to-cell adhesion, leukocyte activation, inflammatory response to antigenic stimulus, cardiac progenitor differentiation, and positive regulation of protein phosphorylation (Figure 5E).

CDs are plasma membrane proteins that mediate cell-to-cell interactions and signal amplification; they are used as cell markers for cell type identification (42, 75). We hypothesized that TMAO upregulates CDs to enhance immune response. The expression changes of 373 CDs from the human protein atlas database were examined. TMAO upregulated 8 genes, including *KLRC1*, *NCAM1*, *CD6*, *CD248*, *MUC1*, *DPP4*, *IL6R*, and *SELE* (Figure 5F). These CDs play important roles in promoting vascular inflammation, cell adhesion, immune response, and signal transduction (Supplemental Table 4). *SELE* is expressed exclusively on ECs and plays a critical role in the immune response and inflammation/rejection during allograft transplantation, and it functions as an innate immune mediator to make ECs act as functional innate immune cells and promote the immune response during transplantation (76–78).

Taken together, these results have demonstrated that TMAO activates and transdifferentiates HAECs into functional innate immune cells and upregulates 24 EC-related genes, 40 cytokines/chemokines/secretomic genes, and 8 CDs. TMAO-stimulated HAECs significantly expand their secretomic capacity and upregulate CDs to enhance vascular inflammation (Figure 5G).

### TMAO activated the phosphorylation of 12 EC-activation and TI-promoting kinases in cytosol, which were integrated with PERK pathways, and PERK inhibitors suppressed TMAO-upregulated ICAM-1.

**Figure 6:**
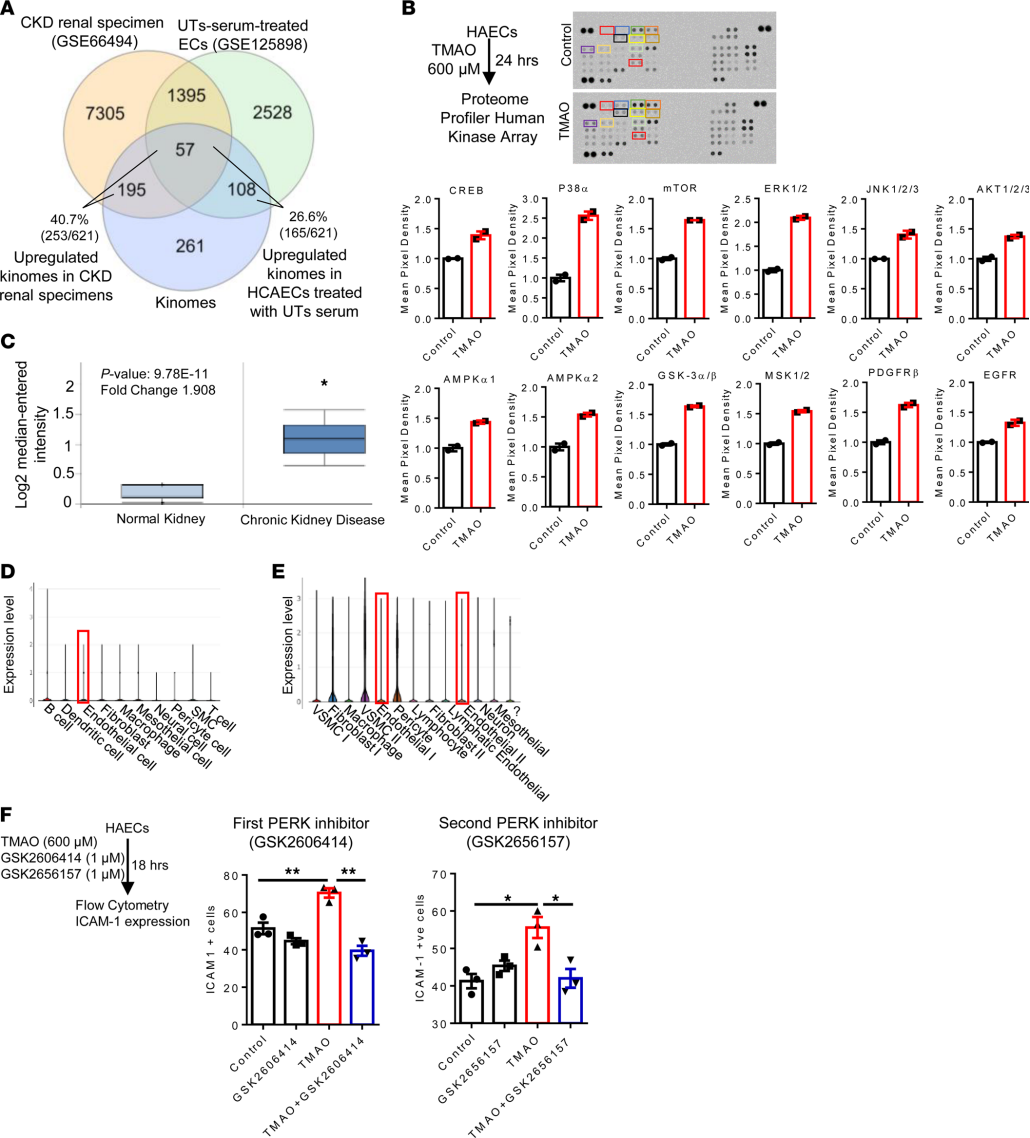
TMAO activated the phosphorylation of 12 kinases, which were integrated with PERK pathways, and PERK inhibitor suppressed TMAO-upregulated ICAM-1. (**A**) CKD renal specimen upregulated 40.6% and UT serum upregulated 26.6% kinomes; *P* < 0.05. (**B**) Human phosphokinase array was performed following the manufacturer’s instructions. HAECs were treated with TMAO for 24 hours. Protein was pooled from 3 wells (*n* = 2). TMAO activated the phosphorylation of 12 kinases. The variations of the manufacturer’s designated positive control (PC) spots between each array were used to determine the CI of nonspecific variations between samples. (**C**) PERK (EIF2AK3) expression in 61 CKD kidneys from the human microarray data set (Nephroseq), FC = 1.9 and *P* = 9.78 × 10–11. (**D** and **E**) PERK expression in different cells of the ascending aorta of HFD mice and human thoracic aorta. The data mining analyses were performed on the scRNA-Seq database of the Broad Institute of MIT and Harvard. (**F**) ICAM-1 expression in HAECs treated with TMAO and 2 PERK inhibitors (GSK2606414 and GSK2656157) were quantified using flow cytometry. The quantitative data of the ICAM-1^+^ cell in each group is presented (*n* = 3). The experiment was repeated 3 times. Data are represented as the mean ± SEM (*t* test; *P < 0.05, **P < 0.01).

Since PERK is a TMAO receptor (25, 52, 53, 61, 79), multiple kinase pathways can serve as signaling mechanisms for TMAO functions in HAECs. Therefore, we determined whether kinome genes are upregulated by CKD pathologies. In CKD renal specimens and UT serum–stimulated HCAECs, 253 (40.7%) and 165 (26.6%) of 621 kinomic genes, respectively, were significantly upregulated (Figure 6A). TMAO binds to PERK in the lumen of ER, and PERK has 4 protein domains, N-terminal signaling peptide, quinonprotein alcohol dehydrogenase-like domain, transmembrane domain, and cytosolic C-terminal protein kinase core domain (80). Thus, we hypothesized that TMAO activates HAECs via binding to PERK at the lumen of ER and via signaling through PERK’s C-terminal cytosolic kinase domain to connect ER stress to cytosolic signaling pathways. To examine this hypothesis, a proteome profiler human phospho-kinase array was used to detect TMAO-induced kinome activities. TMAO increased 12 kinase activities (32.4%), including p38α, ERK1/2, JNK1/2/3, GSK-3, EGFR, MSK, AMPKa1, AKT1/2/3, mTOR, CREB, AMPKα2, and PDGF-Rβ (Figure 6B). Of note, MAPKs promote inflammatory responses (81) and activate glycolysis (82). Furthermore, we examined the subcellular locations of the TMAO-activated kinases from protein subcellular location database COMPARTMENTS (https://compartments.jensenlab.org/Search) and HPA database (https://www.proteinatlas.org/). Most of these kinases are located intracellularly and in the cytosol (Supplemental Table 5). Therefore, PERK can phosphorylate and activate other kinases, presumably by direct or indirect binding to PERK’s cytosolic C-terminal domain.

To determine whether PERK expression was upregulated in CKD, we examined the Nephroseq database. We found that PERK was significantly upregulated in the CKD kidney (83) compared with normal kidney (FC = 1.908, *P* < 0.01) (Figure 6C). We also examined the mouse and human scRNA-Seq data and found that PERK was expressed in the aortic cells of HFD-fed mouse and human thoracic aorta cells (Figure 6, D and E), suggesting that TMAO/PERK pathways may play significant roles in promoting aortic pathologies. By using the Cytoscape pathway analysis, we found that TMAO-activated kinases were directly or indirectly connected with PERK pathway genes, including *EIF2AK3*, *ATF4*, *CEBPB*, *NFE2L2*, *PPP1R15A*, and *FOXO1* (25, 84, 85) (Supplemental Figure 2). To determine whether the PERK pathway is responsible for TMAO-induced ICAM-1 upregulation, we used a PERK inhibitor as a loss-of-function approach. TMAO-induced ICAM-1 upregulation was significantly inhibited by 2 PERK inhibitors, including GSK2606414 and GSK2656157 (25, 51, 86, 87) (Figure 6F). Of note, ICAM-1 expression is not only an EC activation marker, but it also mediates monocyte recruitment, vascular inflammation, and atherosclerosis, since ICAM-1 deficiency in ApoE^–/–^ mice reduces atherosclerotic lesions (88).

Taken together, these results have demonstrated that (a) CKD significantly upregulate the expression of kinomic genes in CKD renal specimens and UT serum–treated HCAECs, suggesting that CKD/UT modulate kinome activities. (b) TMAO activates the phosphorylation and activation of 12 kinases in HAECs, which are integrated with TMAO receptor PERK pathways, suggesting that TMAO not only modulates transcriptome in HAECs, but also posttranslationally modulates the kinome activities in HAECs. Based on subcellular locations and functions of 12 TMAO-activated kinases, these kinases may play significant roles in TMAO-promoted metabolic reprogramming in glycolysis and TCA cycle in addition to EC activation. (c) PERK expression is upregulated in CKD, suggesting that TMAO signaling is activated in CKD, and PERK pathways are responsible for TMAO-induced ICAM-1 upregulation. Finally, (d) significant expression of PERK in mouse and human aortic ECs highlight the roles of the TMAO/PERK pathway in aortic ECs in CVDs.

### TMAO upregulated mitochondrial regulators PLIN4, glycolysis promoter and oxidative phosphorylation (OXPHOS) inhibitor OMA1, and TI promoter OGDHL; downregulated inflammation inhibitor DARS2; and induced mitoROS, while mitoROS inhibitor inhibited TMAO induction of ICAM-1.

**Figure 7:**
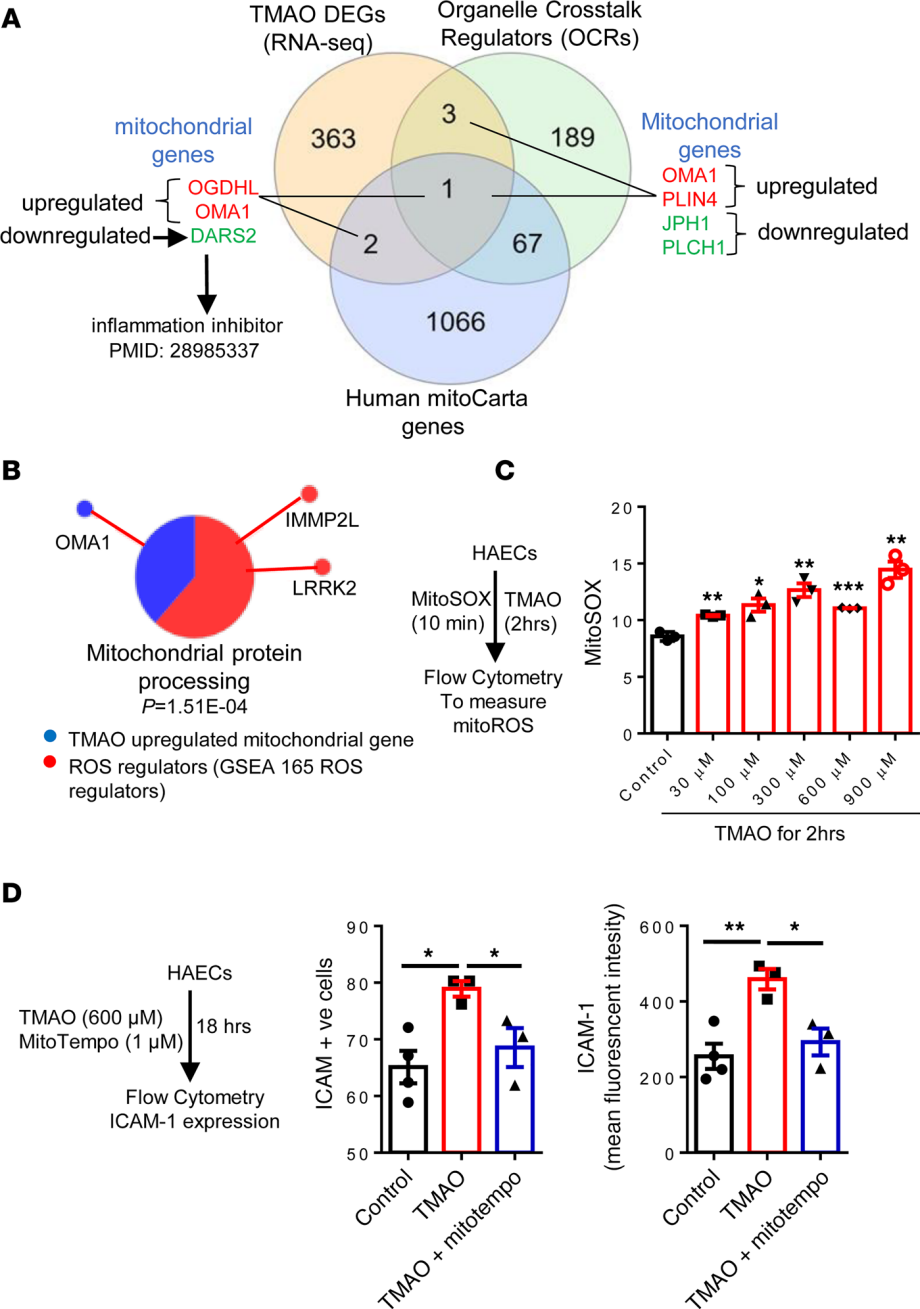
TMAO upregulated *PLIN4*, *OMA1*, and *OGDHL* and downregulated DARS2, and it induced mitoROS and mitoROS inhibitor inhibited TMAO-induced ICAM-1. (**A**) RNA-Seq data show that TMAO significantly modulated the expression of 4 organelle crosstalk regulators (OCRs) with 2 upregulated genes, *PLIN4* and *OMA1*, and 2 downregulated genes, *JPH1* and *PLCH1;* TMAO upregulated 2 MitoCarta genes, *OGDHL* and *OMA1*, and downregulated *DARS2*. (**B**) Cytoscape analysis showed the connection between TMAO-upregulated mitochondrial gene (*OMA1*) and 2 ROS regulators (GSEA). (**C**) TMAO induces mitoROS. HAECs were treated with different TMAO concentrations for 2 hours. Then cells were loaded with MitoSOX, and MitoSOX was detected by flow cytometry (*n* = 3 for each group); the experiment was repeated 3 times. (**D**) Overproduction of mitoROS contributes to TMAO-induced EC activation. HAECs were treated with TMAO (600 μM) and mitoTempo (1 μM) for 18 hours. ICAM-1^+^ cell and mean fluorescence intensity (MFI) were detected using flow cytometry analysis (*n* = 3 for each group; the experiment was repeated 3 times). Data are represented as the mean ± SEM (*t* test; *P < 0.05, **P < 0.01, ***P < 0.001).

Mitochondria are central organelles for ROS generation, immunometabolism, and TI establishment (2–4, 89, 90). We previously reported that lysoPC activate HAECs by inducing mitoROS, and proton leaks in the electron transport chain drives mitoROS generation that can be uncoupled from unchanged ATP synthesis (17, 91–93), suggesting that aortic ECs can be activated when aortic ECs are not damaged and when ATP synthesis is uncompromised. We then hypothesized that TMAO induces mitochondrial stress in HAECs. We determined whether 369 TMAO-modulated genes overlapped with 260 organelle crosstalk regulators (OCRs) and 1,136 human MitoCarta genes (70, 90). We found that 4 OCRs were modulated by TMAO, 2 genes — *PLIN4* and *OMA1* — were significantly upregulated and 2 genes — *JPH1* and *PLCH1* — were downregulated (Figure 7A), which clearly demonstrated the specificity of TMAO modulation of mitochondrial regulators out of all the 260 intracellular OCRs. The *JPH1* downregulation or proteolysis may be triggered by elevated intracellular Ca^2+^ (94).

In addition, TMAO significantly modulate the expression of 3 MitoCarta genes with 2 upregulated genes — *OGDHL* and *OMA1* (responsible for cleavage of mitochondrial inner membrane fusion GTPase OPA1) (95) — and 1 downregulated gene, *DARS2* (inflammation inhibitor) (96). TMAO-upregulated *PLIN4*, also upregulated in brain by fasting, plays a key role in intracellular trafficking of lipids (97), lipid droplet formation, and adipocyte differentiation (98). *PLIN4* was upregulated in rotator cuff injury synovium and was functionally related to oxidative stress and chronic inflammation (99). *OGDHL* was upregulated by a phosphorylation-dependent transcription factor CREB (100). Increased proton efflux due to increased glycolysis facilitates succinate efflux (101), and it triggers the plasma membrane succinate receptor (G protein–coupled receptor 91 [GPR91]) and enhances immunity (102). High expression of GPR91 and succinate trigger intracellular calcium mobilization. Together with TLR ligands, GPR91 senses immunological danger from succinate accumulation in TCA cycle and promote production of proinflammatory cytokines, such as IL-1β (103). OMA1 zinc metallopeptidase activation, with OMA1 inhibitor p32/C1QBP deficiency, results in cleavage of the key mitochondrial fusion GTPase OPA1, and it leads to mitochondrial fragmentation and swelling. Consequently, *OMA1* activation decreases mitochondrial respiration and lipid utilization, sensitizes cells to mitochondrial stress, and triggers a metabolic shift from OXPHOS to glycolysis (95).

Then, we determined whether TMAO-upregulated MitoCarta genes, organelle-mitochondrial crosstalk regulator *OMA1*, and ROS regulators were connected. We used Sytoscape and found that *OMA1* and ROS regulators *IMMP2L* and *LRRK2* were functionally connected and involved in the mitochondrial protein processing pathway (Figure 7B). Mitochondria are functionally and physically coupled to the ER through mitochondria-ER contacts (MERCs), and calcium influx drives mitoROS generation (17, 90, 104). TMAO of 30 μM and higher significantly induced mitoROS generation (Figure 7C). The addition of mitoROS inhibitor mitoTEMPO significantly inhibited TMAO-induced ICAM-1 upregulation (Figure 7D), suggesting that TMAO-induced mitoROS mediate ICAM-1 upregulation and HAEC activation. Of note, we previously reported that inhibition of ICAM-1 in HAECs with mitoTEMPO in vitro were well correlated with functional inhibition of EC activation by mitoTEMPO, including decreased leukocyte rolling on endothelium and decreased monocyte recruitment into mouse aorta in vivo.

These results demonstrate that TMAO–ER stress mediator PERK pathway upregulates the expression of mitochondrial regulators *PLIN4*, *OMA1*, and *OGDHL*; downregulates *DARS2*; and induces mitoROS generation and mitochondrial stress, which mediate ICAM-1 upregulation and HAEC activation.

### CKD PAMP β-glucan and TMAO induced TI; TMAO induced immune metabolic reprogramming, including increased acetyl-CoA generation, increased glycolysis, and proton efflux rates; and glycolysis inhibitor 2-deoxyglucose suppressed TMAO induced ICAM-1 expression.

**Figure 8:**
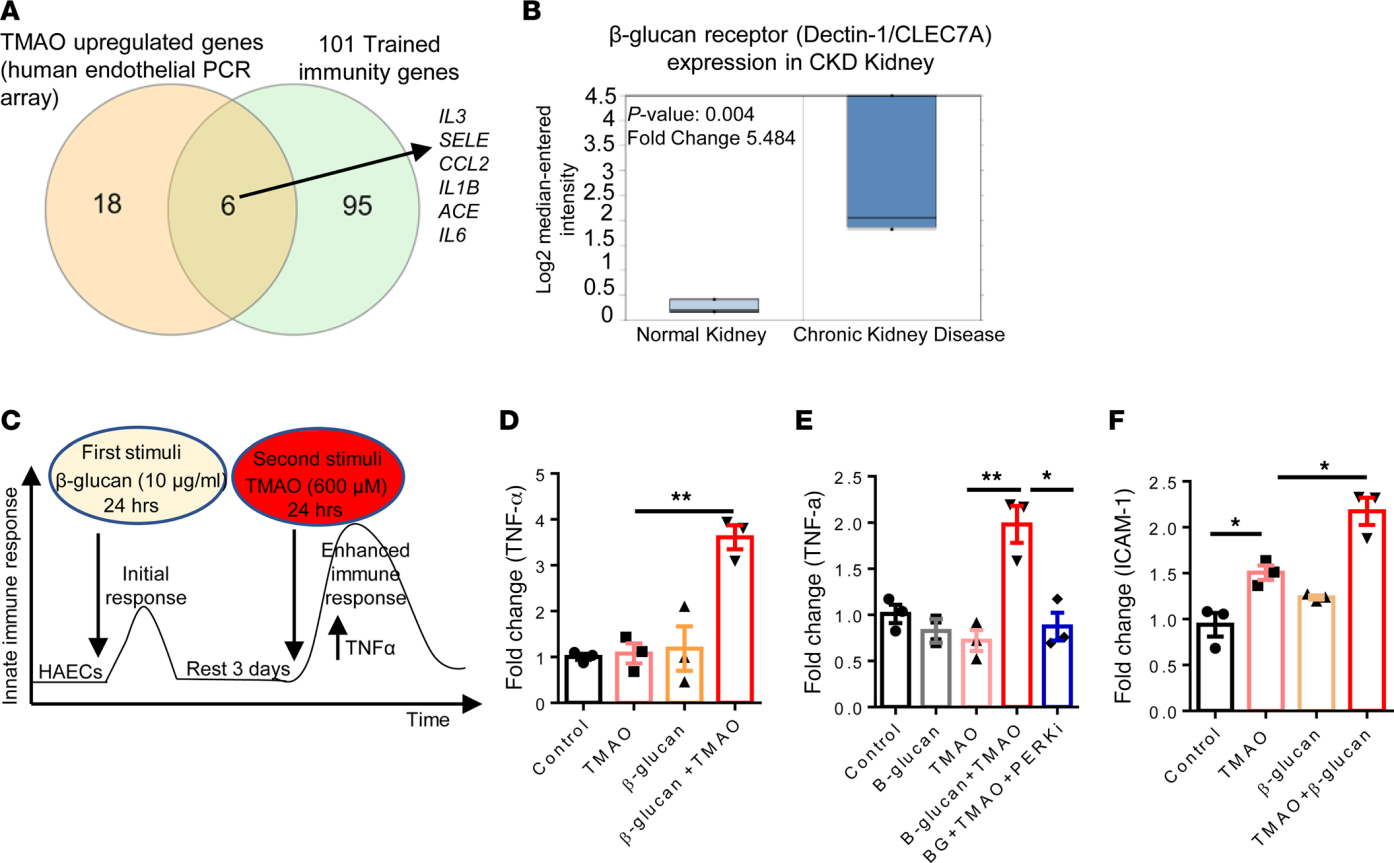
β-Glucan and TMAO induced trained immunity in HAECs. (**A**) TMAO (EC PCR array) upregulated 6 trained immunity–related genes. (**B**) Dectin-1/*CLEC7A* expression in 61 CKD kidneys (microarray data). FC = 5.48, *P* = 0.004. (**C**) Trained immunity experimental design. HAECs were primed with β-glucan (10 μg/mL) for 24 hours, rested for 3 days, and restimulated with TMAO (600 μM). Real-time PCR was used to detect TNF-α expression. (**D** and **F**) TMAO increased ICAM-1 expression (**F**) and TNF-α expression (**D**) after priming with β-glucan. (**E**) PERK inhibitor reduced TNF-α expression (*n* = 3); each experiment was repeated 3 times. Data are represented as the mean ± SEM. (*t* test; *P < 0.05, **P < 0.01).

We recently proposed a concept that ROS create an integrated metabolic sensor network for metabolic stresses and metabolic homeostasis (89) and that lysoPC induce TI (2, 19) gene upregulation (18). In addition, the PERK/ATF4 pathway drives Warburg metabolism and glycolysis (105–107). Therefore, we hypothesized that TMAO induces TI in HAECs via promotion of immune metabolic remodeling. Our PCR array data show that TMAO significantly upregulated 6 TI genes — *IL-3*, *SELE*, *CCL2*, *IL-1β*, *ACE*, and *IL-6* (Figure 8A). β-Glucan is a potent inducer of TI (2, 19). β-Glucan was increased in CKD patients to play roles in promoting inflammation (108). Our data show that the receptor for β-glucan, dectin-1 (*CLEC7A*) (4, 109), was significantly upregulated in CKD renal specimens (Figure 8B), which demonstrated the potential roles of the β-glucan/dectin-1 pathway in promoting inflammation in CKD.

We used a well-established TI in vitro model (2) and stimulated HAECs with β-glucan (10 μg/mL) as the first stimuli for 24 hours, followed by resting for 3 days, and TMAO (600 μM) for 24 hours as the second stimuli (Figure 8C). We found that priming with β-glucan and resting for 3 days followed by restimulation with TMAO significantly increased ICAM-1 and TNF-α expression (Figure 8, D and F), and PERK inhibitor significantly reduced TNF-α (Figure 8E). It has been reported that increased TNF-α was associated with increased ICAM-1 expression in ECs, epithelial cell, and SMCs (Supplemental Table 6). Taken together, these data suggest that TMAO “second stimuli,” working with β-glucan priming, induces TI in HAECs, which further sustains activation status of HAECs and enhances vascular inflammation, whereas PERK inhibition reduces TNF-α and decreases TI.

**Figure 9:**
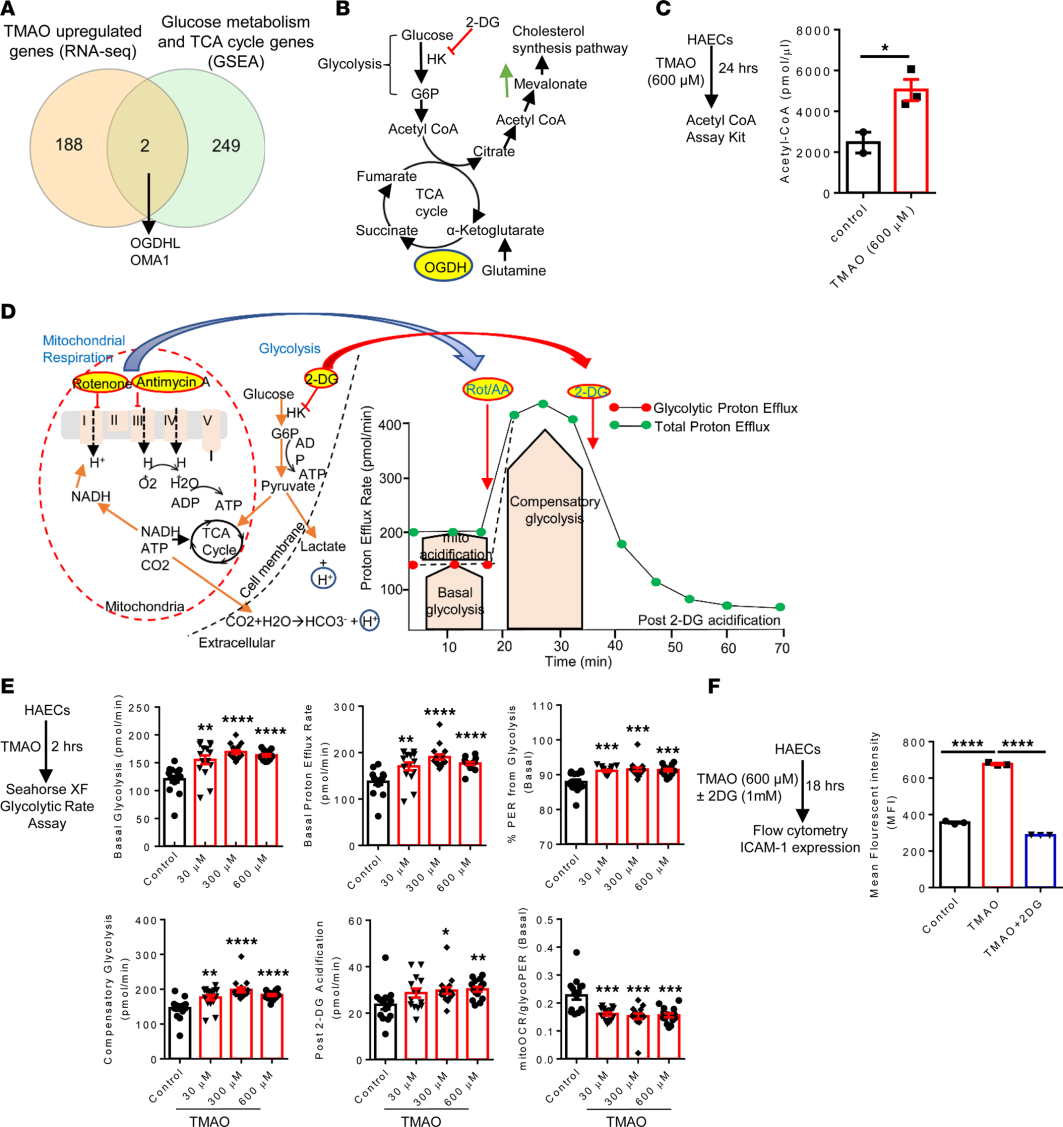
TMAO induced immune metabolic reprogramming — including increased acetyl-CoA, glycolysis, and proton efflux rates — and glycolysis inhibitor suppressed TMAO-induced ICAM-1 expression. (**A**) TMAO upregulated 2 glucose metabolism/TCA cycle genes (*OGDHL* and *OMA1*). (**B**) *OGDHL* is a key step in the TCA cycle and glycolysis pathways. (**C**) TMAO increased acetyl-CoA generation. HAECs were treated with TMAO (600 μM) for 24 hours, and acetyl CoA production was detected using acetyl-CoA assay kit (*n* = 3) following the manufacturer’s instruction. (**D**) The principle and profile of Seahorse glycolytic rate assay. In the cells, energy is produced by 2 different pathways, including mitochondrial respiration and glycolysis. In the glycolysis pathway, glucose is converted into lactate, and the protons are extruded into the extracellular media and detected as extracellular acidification rate (ECAR). In addition, CO_2_ produced by the mitochondrial TCA cycle extruded into the extracellular space and increases ECAR. First, the inhibition of mitochondrial complex I and III by rotenone and antimycin A (Rot/AA) resulted in the reduced rate of proton efflux from respiration, which is calculated and removed from the total proton efflux rate results in glycolytic proton efflux rate (glycoPER). Second, glycolysis inhibitor 2-DG (hexokinase, HK2 inhibitor) is injected to stop glycolytic acidification and confirm pathway specificity. (**E**) Our experimental design (*n* = 16 wells/group). The 6 glycolysis parameters were measured in this assay, including basal glycolysis, basal proton efflux rate, percentage of PER from glycolysis, compensatory glycolysis, post–2-DG acidification, and mitoOCR/glycoPER. (**F**) Inhibition of glycolysis reduced TMAO-induced EC activation (*n* = 3; the experiment was repeated 3 times). Data are represented as the mean ± SEM (*t* test; *P < 0.05, **P < 0.01, ***P < 0.001, ****P < 0.0001).

The 3 metabolic pathways including increased glycolysis, increased acetyl-CoA generation, and increased mevalonate generation contribute to establishment of TI (3, 18, 20, 110). Our results show that TMAO induced 2 mitochondrial genes, *OGDHL* and *OMA1* (Figure 9, A and B). *OGDHL* converts 2-oxoglutarate to succinyl-CoA and CO_2_ in the TCA cycle in mitochondria. Thus, OGDHL promotes TI in the following 3 pathways: (a) by increasing glutaminolysis (2, 111); (b) by increasing fumarate accumulation, which integrates immune and metabolic circuits to induce monocyte epigenetic reprogramming by inhibiting KDM5 histone demethylases (2, 111); and (c) by increasing succinate accumulation (112), which promotes glycolysis (113, 114) and contributes to mitoROS generation (101).

Next, we examined the acetyl-CoA generation and glycolysis in TMAO-treated HAECs. TMAO significantly increased acetyl-CoA generation (Figure 9C), basal glycolysis, basal proton efflux rates (PER), percentages of PER from glycolysis, and compensatory glycolysis (Figure 9, D and E). Moreover, TMAO concentration at 300 μM and 600 μM (115) increased post–2-deoxy-glucose (post–2-DG) acidification rates, which were correlated with TMAO induction of mitoROS. However, the ratios of mitoOCR/glycoPER were significantly decreased, which were well correlated with increased percentages of PER from glycolysis. Although mitochondrial proton cross-inner membrane efflux rates can be used for measuring mitochondrial functions optimally, these results have demonstrated that TMAO-induced proton efflux rates across plasma membrane into the extracellular space are produced more by cytosolic glycolysis than mitochondrial metabolism. Of note, TMAO-increased proton efflux due to increased glycolysis may facilitate succinate efflux (101) derived from TMAO-induced OGDHL promoted succinate accumulation, and it may trigger the plasma membrane succinate receptor GPR91 and enhances immunity (102). Furthermore, glycolytic key enzyme hexokinase (HK2) is mainly bound to the outer mitochondrial membrane on the cytosolic side, where it can gain privileged access to newly synthesized ATP, thus increasing efficiency in glucose usage (116).

Next, we examined the EC activation after inhibition of glycolysis. Our results show that TMAO-induced ICAM-1 expression was suppressed by 2-DG (Figure 9F). Our findings on increased EC glycolysis were well correlated with a previous report that increased expression of HK2 is a proinflammatory phenotype only present in patients with symptomatic but not asymptomatic atherosclerosis (117).

**Figure 10:**
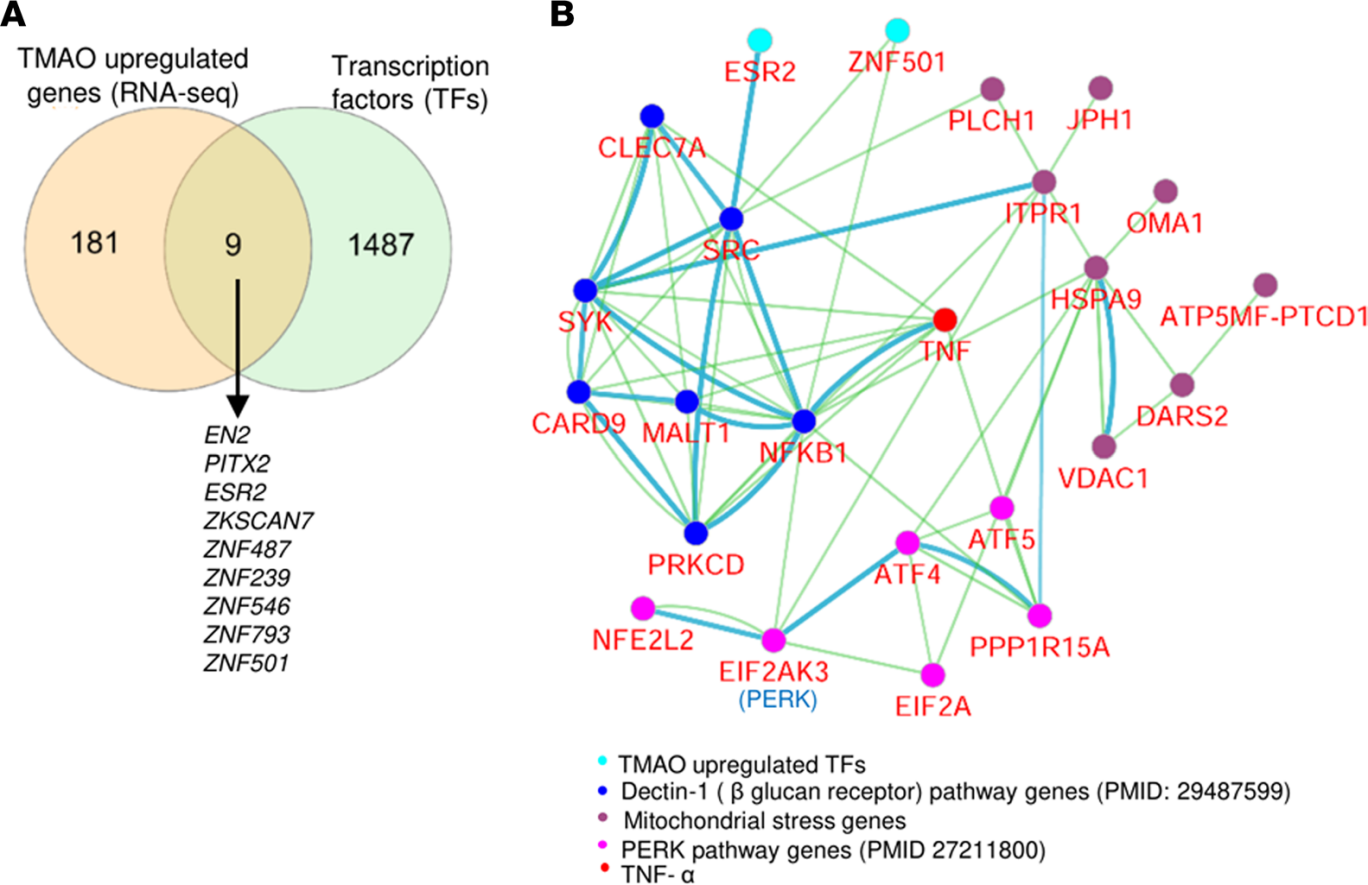
TMAO-upregulated transcription factors were functionally connected to mitochondrial genes, dectin-1 pathway genes, and TNF-α to potentiate immune responses. (**A**) TMAO upregulated 9 transcription factors (TFs). (**B**) Cytoscape pathway analysis showed the network connection between Dectin-1 pathway genes, TNF-α, PERK pathway genes, mitochondrial stress genes, and TMAO-upregulated TFs.

We further performed Cytoscape analysis and found that 12 TMAO-activated kinases were connected to PERK pathway genes and 71 glycolysis pathway genes (18) (Supplemental Figure 3). Previous reports showed that the TMAO-activated kinases can promote the glycolysis and EC activation/dysfunction (Supplemental Table 7). Furthermore, TMAO upregulated 9 transcription factors (TFs) (Figure 10A) that connected to mitochondrial genes, dectin-1 pathway genes, and TNF-α; these connected genes were involved in the immune system regulation and immune response pathways (Figure 10B and Supplemental Figure 4).

Taken together, these results have demonstrated that β-glucan (first stimuli) and TMAO (second stimuli) induce TI, and PERK inhibition reduces TI in HAECs; TMAO increases acetyl-CoA and glycolysis rate; and glycolysis and TI are causative mechanisms underlying TMAO-induced HAEC activation.

## Discussion

ECs are innate immune cells, which are capable of establishing TI. TI is a newly defined quality control criteria for metabolic CVD and CKD risks, infectious agents, DAMPs, and PAMPs in initiating and promoting various diseases. EC activation plays essential roles in promoting the progression of chronic inflammatory diseases and cancer metastasis (1, 3–5, 40, 118–125). Significant progress has been made in elucidating molecular mechanisms underlying EC activation. However, several important issues remained to be addressed: first, whether TMAO is converted in extrahepatic tissues including aorta; second, whether TMAO-PERK pathway promotes TI in HAECs; and third, whether TMAO stimulates transcriptomic and metabolic reprogramming, ER stress–linked mitochondrial stress, and cytosolic glycolysis to establish TI. We examined those issues and made the following findings: (a) ER stress genes were coupregulated with MitoCarta genes in CKD; (b) TMAO significantly upregulated 190 genes in HAECs; (c) FMO3 was expressed in human and mouse aortic cells, suggesting that TAMO is generated in aorta cells; (d) TMAO transdifferentiated HAECs into innate immune cells; (e) TMAO activated the phosphorylation of 12 kinases, which were integrated with PERK pathways, and PERK inhibitor suppressed TMAO-upregulated ICAM-1; (f) TMAO upregulated 3 mitochondrial regulators and downregulated inflammation inhibitor *DARS2*, and induced mitoROS, while the mitoROS inhibitor inhibited TMAO induction of ICAM-1; and (g) β-glucan and TMAO induced TI in HAECs, while PERK inhibitor reduced TI; TMAO increased acetyl-CoA generation, glycolysis, and PER; and glycolysis inhibitor suppressed TMAO-induced ICAM-1.

Increased plasma TMAO concentrations are associated with high risk of CVDs. TMAO concentrations in hemodialysis patients are 40-fold higher than control subjects (126), with ranges between 54.8 and 133.0 μM (127) and reach up to 1103.1 μM (26). TMAO concentration of 600 μM significantly increased expression of ICAM-1, IL1β, activated NLRP3 inflammasomes, induced EC inflammation, increased intracellular ROS (27, 55), and promoted neointima hyperplasia (128). A previous study showed no association between serum TMAO levels and cardiovascular outcomes in ESRD patients (26). In peripheral artery disease and heart failure, higher TMAO levels were associated with higher mortality or cardiac transplantation (129–131). Therefore, high TMAO level can be found in disease conditions, although this issue can be debatable (26, 129).

TMAO can phosphorylate and activate PERK and function through the PERK pathway in different cell types, including hepatocytes, VSMCs, kidney cells, fibroblast, and mouse hippocampal brain tissues (25, 51, 52, 61, 79). Studies showed that the pathophysiological concentrations of TMAO (50 μM) can phosphorylate and activate PERK (25); however, the pharmacological concentrations (0.1M–2M) can reduce ER stress (132). Furthermore, PI3K blockade inhibited Akt/β-catenin signaling and increased FoxO1-mediated TLR4–driven local inflammation (133). However, EC-specific deletion of FoxO1 prevents obesity-related disorders by increasing vascular glycolysis, proliferation, and growth (134). Our results show that TMAO promotes EC glycolysis and TI via PERK pathways, which may not act via the FoxO1 pathway.

Five pathways have been reported to be related to PERK ER stress–induced apoptosis, including PERK/ATF4, PERK/calcineurin, PERK/eIF2α/TDAG51, PERK/eIF2α/IAP2, and PERK/NRF2 (135). However, our results show that TMAO drives TI in HAECs with transcriptomic and posttranslational kinomic approaches via (a) the PERK kinase domain to connect ER stress to cytosolic kinome–driven metabolic reprogramming such as increased glycolysis and (b) calcium influx through ER-mitochondrial tethering complex VAPB-PTPIP51 to trigger mitoROS and calcium plus CREB-, ATF4-, and FoxO1-mediated transcriptomic pathways (25) to promote mitochondrial stress and TCA reprogramming for TI (135–137).

LPS and IL-17 activates HAECs via activating MAPKp38, MAPK-ERK1/2, and MAPK-JNK (123, 125). DAMP receptors activate downstream TFs including NF-κB, AP-1, and CREB (81). CREB is activated by ER stress and promotes upregulation of IL-2, IL-6, and TNF-α (138). PERK can activate and phosphorylate CREB (86), and depletion of CREB decreases PERK expression (139). LPS stimulation and high glucose concentration augment glucose incorporation and glycolytic capacity with the induction of PDGF-β (140). mTOR is activated by insulin (141), and mTOR and AKT signaling control macrophage metabolism and activation (142). In the context of insulin resistance, AKT signaling is affected, resulting in sustained activation of mTORC1 and enhanced glycolysis (143). mTOR-dependent oxidative stress in cytosol promotes oxLDL-induced TI in human monocytes (37).

**Figure 11:**
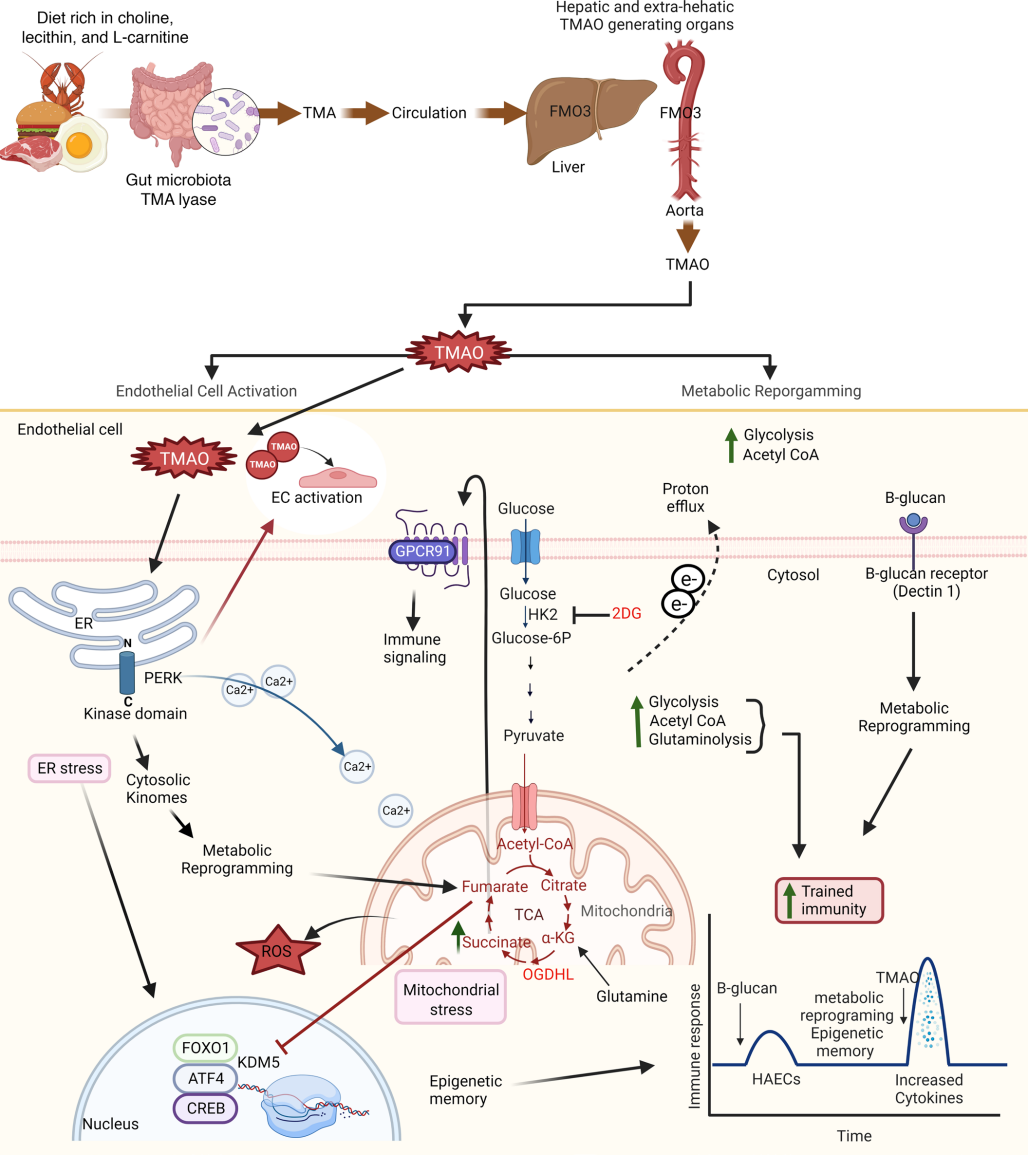
Our working model. Schematic figure showed that TMA is generated by gut microbiota from choline-, lecithin-, and L-carnitine protein–rich diet and is reabsorbed into blood circulation via the portal vein. Then, TMA goes to the liver and other organs, including the aorta, where it can be oxidized by FMO3 to generate TMAO. TMAO binds to the PERK receptor in the ER lumen. PERK connects ER stress in the lumen of ER via its cytosolic kinase domain to cytosolic kinome, CREB, FoxO1, and ATF4 to induce cytosolic stress, mitochondrial stress, and metabolic reprogramming, including increased glycolysis, acetyl-CoA generation, OGDHL-driven glutaminolysis, succinate accumulation, and fumarate accumulation, leading to OMA1-driven mitochondrial fragmentation and switches OXPHOS to glycolysis to establish trained immunity. Created with BioRender.com.

Based on our results, we proposed a new working model (Figure 11). TMAO generation occurs in liver and ECs of other tissues, including aortas, and this generation promotes vascular inflammation in aorta and many tissues. The significance of this finding is that, when TMAO concentrations in blood circulation are decreased after hemodialysis in some patients with CKD/ESRD (144, 145) or when TMAO concentrations are not elevated in CVD, local generation of TMAO in aortic diseases can still promote EC activation and vascular inflammation. PERK serves as a potentially new type of conditional DAMP receptor for TMAO and activates HAECs via PERK pathways. TMAO activated and transdifferentiated HAECs into innate immune cells with upregulation of adhesion molecules, cytokines/chemokines, secretomic genes, and CDs. PERK connects ER stress in the lumen of ER, via its cytosolic kinase domain, to cytosolic kinome and CREB, FoxO1, and ATF4 to cytosolic stress, mitochondrial stress, and metabolic reprogramming to establish TI. Therefore, TMAO-induced metabolic reprogramming includes increased glycolysis, increased acetyl-CoA generation, OGDHL-driven glutaminolysis, succinate accumulation, fumarate accumulation, and OMA1-driven mitochondrial fragmentation and switch OXPHOS to glycolysis (2, 18–20, 110).

Our results have provided insights regarding the roles of gut microbiota–generated TMAO in activating and transdifferentiating ECs into innate immune cells, as well as establishing TI for enhanced proinflammatory cytokine and vascular inflammation and establishing new therapeutic targets for the future development of therapeutics for CVDs and CKDs, inflammations, transplantation, and cancers.

## Methods

### HAEC culture.

The HAECs were obtained from Lonza (CC2535) and cultured in M199 medium (Hyclone Laboratories) supplemented with 20% FBS (HyClone), endothelial cell growth supplement (ECGS; BD Biosciences), heparin (MilliporeSigma) and 1% penicillin, streptomycin, and amphotericin (PSA; Invitrogen).

### Human EC biology PCR array.

Total RNA was isolated using the RNeasy Mini Kit (Qiagen). RNAs were converted to cDNAs with the RT^2^ First Strand Kit (SABiosciences). The expression changes of 84 EC-related genes were detected using Human EC PCR array kit (PAHS-015ZD; Qiagen) following the manufacturer’s instruction.

### qPCR.

RNAs from HAECs and aorta/liver were isolated using the miRNeasy Mini Kit (217004; Qiagen). The cDNA was synthesized using High-Capacity cDNA Reverse Transcription Kit (4368814; Applied Biosystems), and qPCR was performed with iTaq Universal SYBR Green Supermix (Bio-Rad). Results were calculated using the ΔΔCt method relative to the reference control gene β-actin. The sequences of qPCR primers used are summarized in Supplemental Table 8.

### Western blotting.

Protein extracts were collected from HAECs. Protein concentrations were determined by bicinchoninic acid (BCA) assay kit (Thermo Fisher Scientific) with BSA standards. Protein was separated on SDS-PAGE gels and transferred onto PVDF membranes (Bio-Rad) at 400 mA for 90 minutes. Membranes were blocked with 5% (w/v) nonfat milk (Lab Scientific) TBST for 1 hour and incubated with primary antibodies including anti–ICAM-1 antibody (4915; Cell Signaling Technology) and anti–β-Actin antibody (A5441; MilliporeSigma) overnight at 4°C. After membranes were washed for 15 minutes in TBST 3 times, horseradish peroxidase–labeled secondary antibodies were added and incubated at room temperature for 1 hour. Membranes were washed and incubated with enhanced chemiluminescence (ECL) substrate (Pierce, Thermo Fisher Scientific). The expression levels of proteins as indicated by the ECL intensity were measured with ImageJ software (NIH).

### Flow cytometry.

For ICAM-1 measurement, cells were treated with TMAO (600 μM; Sigma Aldrich, 317594), MitoTEMPO (1 μM; Enzon, ALX430-150-M005) (122), GSK2606414 (1 μM; TOCRIS, 5107), or GSK2656157 (1 μM; MilliporeSigma, 5.04651.0001) (146) for 18 hours. After washing with HBSS (Corning) supplemented with 2% FBS (GE Life Sciences), cells were collected and stained with anti–human CD54 antibody (BD Biosciences, 559771) for 30 minutes at room temperature. Next, cells were collected and subjected to flow cytometry analysis.

For mitoROS measurement, HAECs were treated with TMAO for 2 hours and were then stained with MitoSOX (5 μM; Invitrogen, M36008) for 10 minutes and subjected to flow cytometry.

### HAECs training experiments.

Cells were treated with 10 μg/mL β-glucan (provided by David L. Williams at East Tennessee State University) for 24 hours; they were then washed with PBS and cultured for 3 days. Cells were restimulated with TMAO for 24 hours. After 24 hours, cells were collected and lysed in Qiazol to detect TNF-α and ICAM-1 mRNA expressions using qPCR.

### Seahorse XF96 analyzer.

Seahorse XF96 analyzer (Agilent-Seahorse Bioscience) was used to assess 6 glycolysis parameters, including basal glycolysis, compensatory glycolysis, basal PER, percentage of PER from glycolysis, mitochondrial oxygen consumption rate mitoOCR/glycoPER, and post–2-DG acidification according to the manufacturer’s instruction.

### RNA-Seq.

Total RNA libraries were prepared by using Pico Input SMARTer Stranded Total RNA-Seq Kit (Takara). In short, 10 ng total RNA was reverse transcribed via random priming and reverse transcriptase. Full-length cDNA was obtained with SMART (Switching Mechanism At 5′ end of RNA Template) technology. The template-switching reaction was used to keep the strand orientation of the RNA. The ribosomal cDNA was hybridized to mammalian-specific R-Probes and cleaved by ZapR. Libraries containing Illumina adapter with TruSeq HT indexes were subsequently pooled and loaded to the Hiseq 2500. Single-end reads at 75 bp with 30 million reads per sample were generated for bioinformatic analysis. All original RNA-Seq data were deposited in the NCBI’s Gene Expression Omnibus (GEO) database (GSE216833).

### Human phospho-kinase array.

A Proteome human phospho-kinase array kit (ARY003C; R&D Systems Inc.) was used to measure the relative phosphorylation of 37 signaling molecules following the manufacture’s instruction.

### Acetyl CoA measurements.

Acetyl-CoA concentrations were determined with an acetyl-CoA assay kit (ab87546; Abcam) following manufacturer’s instructions.

### Statistics.

Data were expressed as the mean ± SEM. Two-tailed Student’s *t* test was used for statistical comparison between 2 groups. One-way ANOVA with Tukey test was used for multiple comparisons. The Benjamini-Hochberg procedure was employed to control the FDR at 5% to identify differentially expressed genes. Data shown were representatives of 2–3 independent experiments. *P* < 0.05 was considered statistically significant.

### Study approval.

All animal experiments were performed in accordance with the *Guide for the Care and Use of Laboratory Animals* (National Academies Press, 2011) and were approved by the IACUC of Temple University Lewis Katz School of Medicine.

## Author contributions

FS carried out the data gathering and analysis and prepared the tables and figures. LL, KX, RC, Y Shao, YL, Y Sun, NWS, SW, LY, YZ, DLW, CL, LM, RIVP, HZ, XJ, and HW aided with data analysis. XY supervised the experimental design, data analysis, and manuscript writing. All authors read and approved the final manuscript.

## Supplementary Material

Supplemental data

## Version 1 11/17/2022

In-Press Preview

## Version 2 01/10/2023

Electronic publication
