# Supplementary material for: Aorta- and liver-generated TMAO enhances trained immunity for increased inflammation via ER stress/mitochondrial ROS/glycolysis pathways
Source: JCI Insight. 2023 Jan 10;8(1):e158183. doi: 10.1172/jci.insight.158183 (PMC9870092; doi:10.1172/jci.insight.158183)
Supplement: Supplemental data [file jciinsight-8-158183-s168.pdf]

Aorta- and liver-generated TMAO enhances trained immunity for increased inflammation via ER stress/mitochondrial ROS/glycolysis pathways

Fatma Saaoud[1], Lu Liu[2], Keman Xu[1], Ramon Cueto[2], Ying Shao[1], Yifan Lu[1], Yu Sun[1], Nathaniel W. Snyder[2], Sheng Wu[2], Ling Yang[3], Yan Zhou[4], David L. Williams[5], Chuanfu Li[5], Laisel Martinez[6], Roberto I Vazquez-Padron[6], Huaqing Zhao[7], Xiaohua Jiang[1, 2], Hong Wang[2], Xiaofeng Yang[1, 2, #]

[1]Centers of Cardiovascular Research, [2]Metabolic Disease Research and Thrombosis Research Center, Departments of Cardiovascular Sciences, Biomedical Education and Data Sciences, [3]Department of Medical Genetics and Molecular Biochemistry, Temple University Lewis Katz School of Medicine, Philadelphia, PA; [4]Biostatistics and Bioinformatics Facility, Fox Chase Cancer Center, Temple Health, Philadelphia, PA; [5]Department of Surgery, Center of Excellence in Inflammation, Infectious Disease and Immunity, Quillen College of Medicine, East Tennessee State University, Johnson City, TN; [6]DeWitt Daughtry Family Department of Surgery, Leonard M. Miller School of Medicine, University of Miami, Miami, FL; [7]Center for Biostatistics and Epidemiology, Temple University Lewis Katz School of Medicine, Philadelphia, PA.

#All the correspondences should be addressed: Xiaofeng Yang, MD, PhD, FAHA, Department of Cardiovascular Sciences, Lewis Katz School of Medicine at Temple University, 3500 North Broad Street, Philadelphia, PA 19140, Email: xfyang@temple.edu

Keywords: trimethylamine N-oxide (TMAO), PERK, trained immunity, mitochondrial-ER stress, RNA-Seq

Supplementary data

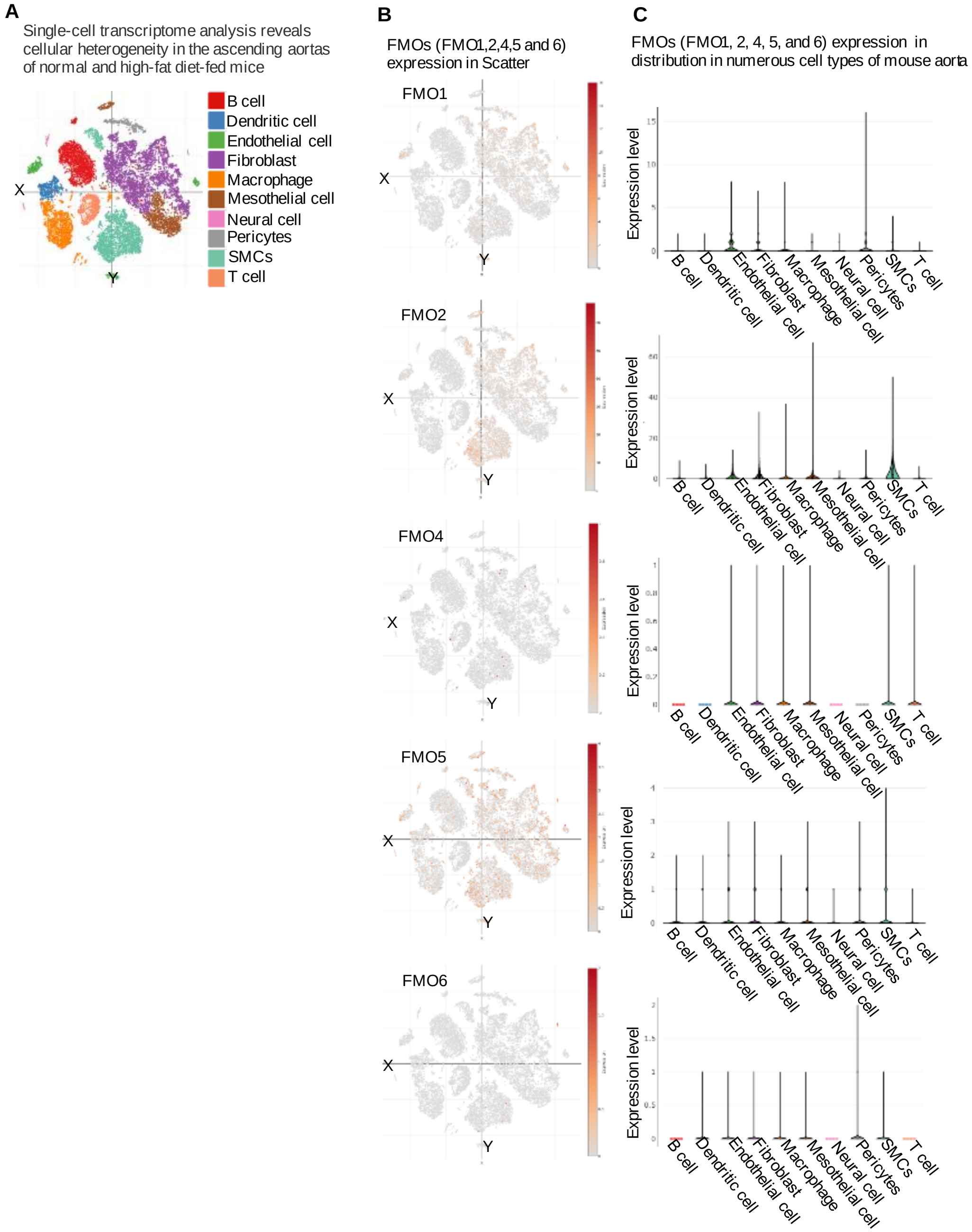

Supplementary figure 1. A. 10 cell types identified in the ascending aorta including endothelial cells (ECs), fibroblasts, vascular smooth muscle cells (SMCs), immune cells (B cells, T cells, macrophages, and dendritic cells), mesothelial cells, pericytes, and neural cells. (B and C). The mRNA transcripts of five types of flavin-containing dimethylaniline monooxygenase FMOs including FMO1, FMO2, FMO4, FMO5, and FMO6

PERK pathway genes were connected to TMAO-activated kinases

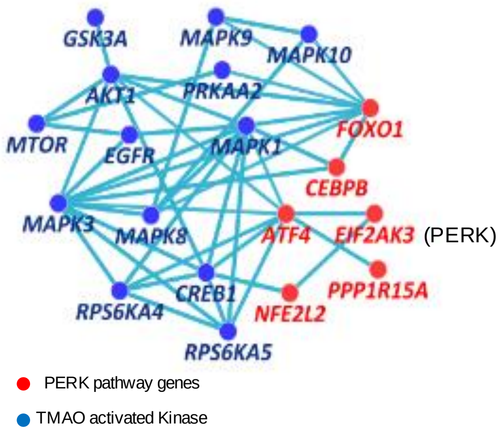

Supplementary figure 2. Cytoscape analysis showed the connection between PERK pathway genes and TMAO-activated kinases

Glycolysis genes directly/indirectly connected to TMAO activated kinases and PERK pathway genes

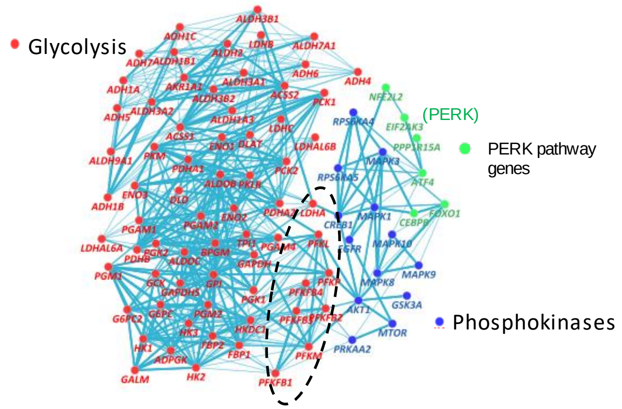

Supplementary figure 3. Cytoscape analysis showed the functional connection between the PERK pathway genes, TMAO-activated kinases, and 71 glycolysis genes

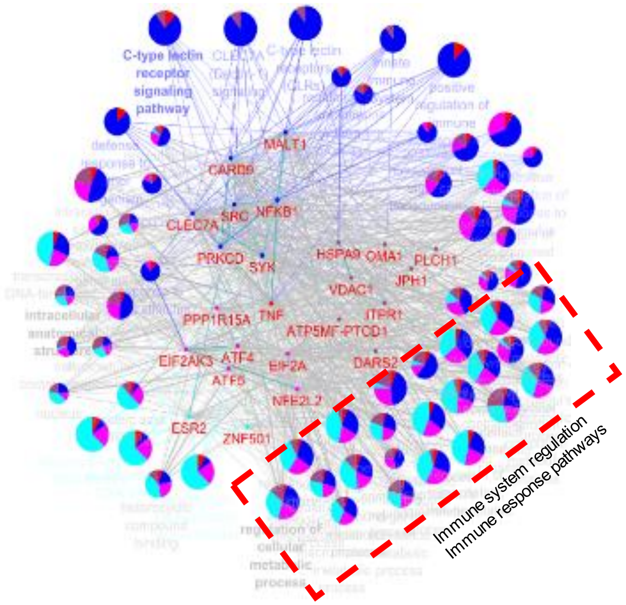

Supplementary Figure 4. Cytoscape analysis showed the network connection between Dectin-1 pathway genes, TNF-α, PERK pathway genes, mitochondrial stress genes, and TMAO-upregulated TFs.

Supplementary Table 1A. Complete list of 137 mitoCarta genes and 17 ER stress genes CKD upregulated in human coronary arterial endothelial cells (HCAECs) treated with uremic toxin (UTs) serum from CKD patients. This gene list is related to Figure 1A.

Gene symbol	logFC	Category	Gene symbol	logFC	Category	Gene symbol	logFC	Category
PNPLA8	3.2	MitoCarta gene	TOMM20L	1.16	MitoCarta gene	PTGES2	0.757	MitoCarta gene
ABCB6	2.59	MitoCarta gene	ABCD3	1.15	MitoCarta gene	SIRT5	0.756	MitoCarta gene
TARS2	2.58	MitoCarta gene	AUH	1.14	MitoCarta gene	COX4I1	0.752	MitoCarta gene
GLDC	2.29	MitoCarta gene	UQCRB	1.14	MitoCarta gene	GSR	0.748	MitoCarta gene
ABCA9	2.18	MitoCarta gene	ARG2	1.13	MitoCarta gene	CA5B	0.744	MitoCarta gene
PDK4	2.08	MitoCarta gene	CMPK2	1.13	MitoCarta gene	PDSS2	0.736	MitoCarta gene
GLS2	2.03	MitoCarta gene	POLG	1.12	MitoCarta gene	CA5A	0.733	MitoCarta gene
SLC25A16	1.99	MitoCarta gene	AGK	1.12	MitoCarta gene	SFXN5	0.714	MitoCarta gene
AADAT	1.98	MitoCarta gene	COQ6	1.12	MitoCarta gene	GPX4	0.705	MitoCarta gene
ATAD1	1.93	MitoCarta gene	NME6	1.07	MitoCarta gene	PDE2A	0.698	MitoCarta gene
ACSL6	1.91	MitoCarta gene	TOMM34	1.06	MitoCarta gene	COX4I2	0.687	MitoCarta gene
ADHFE1	1.82	MitoCarta gene	ACAD11	1.06	MitoCarta gene	LETMD1	0.683	MitoCarta gene
OGDHL	1.79	MitoCarta gene	COQ3	1.06	MitoCarta gene	PHYH	0.676	MitoCarta gene
ACACB	1.74	MitoCarta gene	CRY1	1.06	MitoCarta gene	MFF	0.664	MitoCarta gene
UCP2	1.69	MitoCarta gene	CPT2	1.06	MitoCarta gene	SARS2	0.655	MitoCarta gene
KMO	1.68	MitoCarta gene	COX7A2	1.03	MitoCarta gene	DMPK	0.654	MitoCarta gene
CPS1	1.65	MitoCarta gene	PPM1K	1.02	MitoCarta gene	ECHDC3	0.646	MitoCarta gene
ADCY10	1.63	MitoCarta gene	SLC25A37	1.01	MitoCarta gene	ACADM	0.641	MitoCarta gene
FPGS	1.63	MitoCarta gene	FASTKD1	1.01	MitoCarta gene	NBR1	0.637	MitoCarta gene
SLC25A45	1.62	MitoCarta gene	GLOD4	1	MitoCarta gene	HDHD3	0.632	MitoCarta gene
BCO2	1.62	MitoCarta gene	ACSL1	1	MitoCarta gene	EXOG	0.628	MitoCarta gene
DNA2	1.61	MitoCarta gene	NIF3L1	0.992	MitoCarta gene	TST	0.626	MitoCarta gene
MAOB	1.6	MitoCarta gene	ACSS1	0.982	MitoCarta gene	LARS2	0.624	MitoCarta gene
MUTYH	1.59	MitoCarta gene	MRPL1	0.97	MitoCarta gene	CYP11A1	0.621	MitoCarta gene
AGXT2	1.57	MitoCarta gene	GRPEL2	0.966	MitoCarta gene	MYO19	0.621	MitoCarta gene
MTRF1	1.53	MitoCarta gene	VARS2	0.949	MitoCarta gene	CPT1C	0.615	MitoCarta gene
MRPL47	1.51	MitoCarta gene	DHRS2	0.938	MitoCarta gene	ALAS1	0.614	MitoCarta gene
GLYAT	1.5	MitoCarta gene	STX17	0.918	MitoCarta gene	BAD	0.614	MitoCarta gene
MOCS1	1.45	MitoCarta gene	ME3	0.913	MitoCarta gene	AMT	0.61	MitoCarta gene
NAT8L	1.45	MitoCarta gene	MAOA	0.899	MitoCarta gene	HTATIP2	0.602	MitoCarta gene
NIPSNAP3B	1.44	MitoCarta gene	TBRG4	0.898	MitoCarta gene	HSD17B4	0.601	MitoCarta gene
ABCD2	1.44	MitoCarta gene	ECHDC2	0.889	MitoCarta gene	HINT3	0.593	MitoCarta gene
SLC25A14	1.4	MitoCarta gene	LETM2	0.874	MitoCarta gene	ACP6	0.585	MitoCarta gene
ACOT11	1.37	MitoCarta gene	FAM162A	0.871	MitoCarta gene	BCL2L11	1.4	ER and MitoCarta gene
BDH1	1.37	MitoCarta gene	PIF1	0.857	MitoCarta gene	HTRA2	1.05	ER and MitoCarta gene
BCS1L	1.35	MitoCarta gene	AIFM3	0.854	MitoCarta gene	GANC	2.33	ER stress genes
EHHADH	1.34	MitoCarta gene	SPATA19	0.846	MitoCarta gene	DDIT3	1.98	ER stress genes
NNT	1.34	MitoCarta gene	D2HGDH	0.838	MitoCarta gene	MAPK8	1.93	ER stress genes
SPHK2	1.33	MitoCarta gene	SARDH	0.829	MitoCarta gene	SRF	1.69	ER stress genes
MTRF1L	1.29	MitoCarta gene	MTHFD2L	0.828	MitoCarta gene	SLC17A2	1.36	ER stress genes
PPA2	1.28	MitoCarta gene	RDH14	0.827	MitoCarta gene	ERO1LB	1.1	ER stress genes
SPHKAP	1.27	MitoCarta gene	AASS	0.818	MitoCarta gene	TOR1A	1.01	ER stress genes
TRMT2B	1.27	MitoCarta gene	ACADVL	0.817	MitoCarta gene	EDEM1	0.882	ER stress genes
COQ2	1.27	MitoCarta gene	COQ4	0.801	MitoCarta gene	CASP12	0.84	ER stress genes
AKAP10	1.26	MitoCarta gene	OXR1	0.791	MitoCarta gene	TRIB3	0.658	ER stress genes
ALDH1L1	1.25	MitoCarta gene	ACAA1	0.787	MitoCarta gene	HSPA6	0.657	ER stress genes
GATM	1.25	MitoCarta gene	SFXN3	0.769	MitoCarta gene	CREB3L3	0.647	ER stress genes
MRPS27	1.24	MitoCarta gene	ABAT	0.766	MitoCarta gene	PPP1R15A	0.64	ER stress genes
SLC25A27	1.23	MitoCarta gene	MSRA	0.766	MitoCarta gene	ATF6B	0.638	ER stress genes
IMMP1L	1.21	MitoCarta gene	SLC25A15	0.761	MitoCarta gene	MANF	0.631	ER stress genes
OTC	1.17	MitoCarta gene	IDH3G	0.759	MitoCarta gene			

Supplementary Table 1B. Complete list of 232 mitoCarta genes and 25 ER stress genes upregulated in peripheral blood mononuclear cells (PBMCs) isolated from end-stage renal disease patients. This gene list is related to Figure 1B.

Gene symbol	Log2 FC	Category	Gene symbol	Log2 FC	Category	Gene symbol	Log2 FC	Category	Gene symbol	Log2FC	Category
ACAA1	0.909	MitoCarta gene	GLUD1	0.692	MitoCarta gene	MTHFD2	1.486	MitoCarta gene	SNAP29	1.050	MitoCarta gene
ACAA2	1.799	MitoCarta gene	GOLPH3	0.860	MitoCarta gene	MTIF2	0.915	MitoCarta gene	SND1	0.664	MitoCarta gene
ACADM	1.132	MitoCarta gene	GRPEL1	0.679	MitoCarta gene	NBR1	0.777	MitoCarta gene	SOD1	1.647	MitoCarta gene
ACADVL	0.637	MitoCarta gene	GRSF1	1.151	MitoCarta gene	NDUFA1	2.914	MitoCarta gene	SPHK2	1.031	MitoCarta gene
ACAT1	0.859	MitoCarta gene	GSTK1	0.786	MitoCarta gene	NDUFA13	1.653	MitoCarta gene	SPTLC2	2.129	MitoCarta gene
ACLY	1.271	MitoCarta gene	HADHA	1.233	MitoCarta gene	NDUFA2	0.911	MitoCarta gene	SSBP1	1.386	MitoCarta gene
ACO2	0.834	MitoCarta gene	HIGD1A	2.730	MitoCarta gene	NDUFA4	3.126	MitoCarta gene	STARD7	0.606	MitoCarta gene
AFG3L2	1.047	MitoCarta gene	HINT1	3.877	MitoCarta gene	NDUFA5	3.810	MitoCarta gene	SUCLA2	0.908	MitoCarta gene
AK2	1.484	MitoCarta gene	HSD17B10	1.016	MitoCarta gene	NDUFA6	2.517	MitoCarta gene	TAZ	1.208	MitoCarta gene
ALDH2	1.175	MitoCarta gene	HSD17B4	1.091	MitoCarta gene	NDUFA7	1.457	MitoCarta gene	TFAM	0.913	MitoCarta gene
BAD	1.267	MitoCarta gene	HSPA9	2.320	MitoCarta gene	NDUFA9	0.634	MitoCarta gene	TFB2M	0.931	MitoCarta gene
BCL2A1	5.007	MitoCarta gene	HSPD1	0.677	MitoCarta gene	NDUFAB1	0.691	MitoCarta gene	THG1L	1.066	MitoCarta gene
BID	1.188	MitoCarta gene	HSPE1	1.431	MitoCarta gene	NDUFB1	2.660	MitoCarta gene	TIMM10	0.764	MitoCarta gene
BLOC1S1	0.989	MitoCarta gene	HTATIP2	0.940	MitoCarta gene	NDUFB2	2.003	MitoCarta gene	TIMM17A	1.807	MitoCarta gene
BNIP3L	1.060	MitoCarta gene	IARS2	1.205	MitoCarta gene	NDUFB3	2.195	MitoCarta gene	TIMM44	0.798	MitoCarta gene
C1QBP	1.303	MitoCarta gene	IDE	0.638	MitoCarta gene	NDUFB5	1.244	MitoCarta gene	TIMM8B	2.069	MitoCarta gene
CCDC90B	1.322	MitoCarta gene	IDH3A	0.741	MitoCarta gene	NDUFB7	1.070	MitoCarta gene	TK2	0.780	MitoCarta gene
CHCHD2	1.191	MitoCarta gene	IDI1	1.850	MitoCarta gene	NDUFB8	0.927	MitoCarta gene	TMEM126B	1.829	MitoCarta gene
CHCHD7	1.419	MitoCarta gene	ISCA1	0.896	MitoCarta gene	NDUFC1	1.034	MitoCarta gene	TMEM70	1.469	MitoCarta gene
CMC2	1.586	MitoCarta gene	KMO	0.934	MitoCarta gene	NDUFS1	0.982	MitoCarta gene	TOMM20	1.324	MitoCarta gene
CMC4	2.012	MitoCarta gene	KYAT3	0.872	MitoCarta gene	NDUFS3	0.740	MitoCarta gene	TRIAP1	1.717	MitoCarta gene
COA1	1.005	MitoCarta gene	LAP3	1.154	MitoCarta gene	NDUFS4	1.577	MitoCarta gene	TRMT1	1.625	MitoCarta gene
COMT	2.031	MitoCarta gene	LDHB	0.718	MitoCarta gene	NDUFS6	1.267	MitoCarta gene	TUFM	1.294	MitoCarta gene
COQ2	1.395	MitoCarta gene	LIPT1	1.113	MitoCarta gene	NDUFS7	0.601	MitoCarta gene	UQCR10	1.413	MitoCarta gene
COX11	1.216	MitoCarta gene	LONP1	0.983	MitoCarta gene	NDUFV2	1.844	MitoCarta gene	UQCR11	1.032	MitoCarta gene
COX4I1	1.820	MitoCarta gene	LRPPRC	0.588	MitoCarta gene	NFU1	1.941	MitoCarta gene	UQCRB	4.998	MitoCarta gene
COX5A	0.860	MitoCarta gene	LYPLA1	1.080	MitoCarta gene	NME3	1.672	MitoCarta gene	UQCRC1	0.668	MitoCarta gene
COX5B	1.439	MitoCarta gene	LYRM2	1.765	MitoCarta gene	NNT	0.873	MitoCarta gene	UQCRC2	1.091	MitoCarta gene
COX6A1	1.060	MitoCarta gene	MCUR1	0.890	MitoCarta gene	NUDT2	1.195	MitoCarta gene	UQCRFS1	1.280	MitoCarta gene
COX6C	3.475	MitoCarta gene	ME2	0.737	MitoCarta gene	NUDT9	0.878	MitoCarta gene	UQCRQ	2.668	MitoCarta gene
COX7A2	1.901	MitoCarta gene	METTL17	0.765	MitoCarta gene	OAT	1.476	MitoCarta gene	VDAC2	0.857	MitoCarta gene
COX7A2L	1.081	MitoCarta gene	METTL5	1.723	MitoCarta gene	OGDH	1.372	MitoCarta gene	VDAC3	0.995	MitoCarta gene
COX7B	4.184	MitoCarta gene	MFN1	1.662	MitoCarta gene	PARK7	1.250	MitoCarta gene	VPS13D	0.962	MitoCarta gene
COX7C	4.324	MitoCarta gene	MGST3	0.809	MitoCarta gene	PARL	0.791	MitoCarta gene	YME1L1	0.997	MitoCarta gene
COX8A	0.882	MitoCarta gene	MICU2	1.392	MitoCarta gene	PDP1	0.643	MitoCarta gene	YRDC	1.022	MitoCarta gene
CPT1A	1.142	MitoCarta gene	MIEF1	0.614	MitoCarta gene	PHB	1.068	MitoCarta gene	CRYZ	2.054	MitoCarta gene
CRY1	0.821	MitoCarta gene	MMADHC	1.108	MitoCarta gene	PHYH	1.100	MitoCarta gene	PDHB	1.446	MitoCarta gene
CYC1	1.384	MitoCarta gene	MPC1	0.643	MitoCarta gene	PMAIP1	2.871	MitoCarta gene	SUCLG2	0.889	MitoCarta gene
CYCS	1.517	MitoCarta gene	MRPL11	1.230	MitoCarta gene	PMPCB	0.954	MitoCarta gene	PDHA1	0.805	MitoCarta gene
CYP27A1	1.025	MitoCarta gene	MRPL13	1.766	MitoCarta gene	PPA2	1.002	MitoCarta gene	BAX	2.489	ER and mitoCarta gene
DAP3	0.829	MitoCarta gene	MRPL15	0.714	MitoCarta gene	PPIF	1.328	MitoCarta gene	PFDN5	4.041	ER stress gene
DBI	4.380	MitoCarta gene	MRPL17	1.148	MitoCarta gene	PRDX3	0.918	MitoCarta gene	PPP1R15A	1.909	ER stress gene
DDX28	3.002	MitoCarta gene	MRPL18	0.834	MitoCarta gene	PRDX4	1.309	MitoCarta gene	SERP1	1.782	ER stress gene
DLAT	1.203	MitoCarta gene	MRPL19	1.065	MitoCarta gene	PRDX6	0.885	MitoCarta gene	HSPA5	1.736	ER stress gene
DLD	1.358	MitoCarta gene	MRPL22	2.031	MitoCarta gene	PTRH2	0.803	MitoCarta gene	HSPA4	1.601	ER stress gene
DLST	0.708	MitoCarta gene	MRPL23	1.203	MitoCarta gene	QDPR	0.846	MitoCarta gene	CCT2	1.541	ER stress gene
DNAJC15	3.238	MitoCarta gene	MRPL3	2.129	MitoCarta gene	RFK	1.197	MitoCarta gene	DNAJC10	1.490	ER stress gene
DNM1L	0.694	MitoCarta gene	MRPL33	0.595	MitoCarta gene	RHOT2	1.159	MitoCarta gene	SIL1	1.329	ER stress gene
DUT	0.613	MitoCarta gene	MRPL34	1.211	MitoCarta gene	RIDA	0.737	MitoCarta gene	MAPK9	1.300	ER stress gene
ECHDC1	1.556	MitoCarta gene	MRPL35	1.701	MitoCarta gene	RMDN1	1.197	MitoCarta gene	PDIA3	1.270	ER stress gene
ECI1	1.178	MitoCarta gene	MRPL39	1.258	MitoCarta gene	SCO2	1.390	MitoCarta gene	DNAJB9	1.208	ER stress gene
ELAC2	1.133	MitoCarta gene	MRPL4	2.280	MitoCarta gene	SCP2	1.189	MitoCarta gene	SEC63	1.203	ER stress gene
ENDOG	1.356	MitoCarta gene	MRPL40	2.543	MitoCarta gene	SDHB	1.015	MitoCarta gene	DBF4	1.130	ER stress gene
ETFA	1.134	MitoCarta gene	MRPL42	3.353	MitoCarta gene	SDHC	0.800	MitoCarta gene	UFD1L	1.102	ER stress gene
ETFB	1.207	MitoCarta gene	MRPL48	0.976	MitoCarta gene	SFXN3	2.066	MitoCarta gene	PPP1CC	1.004	ER stress gene
ETFDH	1.160	MitoCarta gene	MRPL57	1.012	MitoCarta gene	SHMT2	0.841	MitoCarta gene	CANX	0.939	ER stress gene
FAM162A	0.701	MitoCarta gene	MRPL58	1.852	MitoCarta gene	SLC25A24	1.050	MitoCarta gene	ATF4	0.930	ER stress gene
FARS2	0.692	MitoCarta gene	MRPL9	0.759	MitoCarta gene	SLC25A28	1.090	MitoCarta gene	PRKCSH	0.790	ER stress gene
FASTK	0.601	MitoCarta gene	MRPS15	2.202	MitoCarta gene	SLC25A32	0.816	MitoCarta gene	YY1	0.744	ER stress gene
FDX1	1.504	MitoCarta gene	MRPS16	1.066	MitoCarta gene	SLC25A46	1.229	MitoCarta gene	DERL1	0.734	ER stress gene
FTH1	1.763	MitoCarta gene	MRPS22	1.191	MitoCarta gene	SLC25A5	0.764	MitoCarta gene	MCM5	0.701	ER stress gene
GADD45GIP1	1.368	MitoCarta gene	MRPS28	1.509	MitoCarta gene	SLC25A6	1.181	MitoCarta gene	HIF1A	0.687	ER stress gene
GHITM	1.750	MitoCarta gene	MRPS30	1.216	MitoCarta gene	SLC30A9	1.048	MitoCarta gene	UBE2G2	0.672	ER stress gene
GLOD4	0.881	MitoCarta gene	MRPS33	2.727	MitoCarta gene	SLIRP	2.912	MitoCarta gene	GANAB	0.625	ER stress gene

Supplementary Table 1C. Complete list of 400 mitoCarta genes and 59 ER stress genes upregulated in renal biopsy specimens collected from CKD patients. This gene list is related to Figure 1C

Gene symbol	log2FC	Category	Gene symbol	log2FC	Category	Gene symbol	log2FC	Category	Gene symbol	log2FC	Category
AADAT	0.655	MitoCarta gene	CCDC127	1.072	MitoCarta gene	FAHD1	0.784	MitoCarta gene	LYRM4	0.820	MitoCarta gene
ABCB10	0.642	MitoCarta gene	CCDC51	0.783	MitoCarta gene	FAM136A	0.604	MitoCarta gene	LYRM7	1.205	MitoCarta gene
ABCD2	0.946	MitoCarta gene	CCDC90B	1.179	MitoCarta gene	FAM162A	0.783	MitoCarta gene	MARS2	1.826	MitoCarta gene
ABCD3	1.228	MitoCarta gene	CHCHD1	0.675	MitoCarta gene	FAM210A	1.223	MitoCarta gene	MCAT	1.925	MitoCarta gene
ABHD10	1.248	MitoCarta gene	CHCHD2	0.701	MitoCarta gene	FAM210B	0.862	MitoCarta gene	MCCC2	0.629	MitoCarta gene
ABHD11	0.936	MitoCarta gene	CHCHD7	0.730	MitoCarta gene	FASTKD2	0.587	MitoCarta gene	MCL1	0.838	MitoCarta gene
ACAA2	0.629	MitoCarta gene	CISD1	0.821	MitoCarta gene	FASTKD5	0.947	MitoCarta gene	MCUB	0.803	MitoCarta gene
ACACA	0.772	MitoCarta gene	CLPP	0.666	MitoCarta gene	FBXL4	0.772	MitoCarta gene	ME2	1.240	MitoCarta gene
ACADM	0.887	MitoCarta gene	CLPX	0.721	MitoCarta gene	FDXR	0.843	MitoCarta gene	METTL15	0.936	MitoCarta gene
ACLY	0.620	MitoCarta gene	CLYBL	0.686	MitoCarta gene	FHIT	0.819	MitoCarta gene	METTL4	0.780	MitoCarta gene
ACOT2	0.977	MitoCarta gene	COA3	0.794	MitoCarta gene	FTH1	0.818	MitoCarta gene	METTL5	0.903	MitoCarta gene
ACSL1	1.195	MitoCarta gene	COA6	0.926	MitoCarta gene	FUNDC1	0.939	MitoCarta gene	METTL8	1.049	MitoCarta gene
ACSL6	1.377	MitoCarta gene	COA7	0.897	MitoCarta gene	FXN	1.001	MitoCarta gene	MFF	0.661	MitoCarta gene
ACSM1	0.951	MitoCarta gene	COQ10B	1.575	MitoCarta gene	GATC	1.094	MitoCarta gene	MGST3	0.860	MitoCarta gene
ACSS3	1.156	MitoCarta gene	COQ2	1.144	MitoCarta gene	GDAP1	1.148	MitoCarta gene	MICU2	0.733	MitoCarta gene
ADCY10	1.281	MitoCarta gene	COX11	0.875	MitoCarta gene	GFER	0.681	MitoCarta gene	MIEF1	0.696	MitoCarta gene
AGK	1.345	MitoCarta gene	COX16	1.163	MitoCarta gene	GFM1	0.944	MitoCarta gene	MIGA1	0.629	MitoCarta gene
AGPAT4	0.930	MitoCarta gene	COX18	0.951	MitoCarta gene	GFM2	0.592	MitoCarta gene	MMAA	0.586	MitoCarta gene
AGPAT5	0.916	MitoCarta gene	COX20	0.671	MitoCarta gene	GLOD4	1.033	MitoCarta gene	MMADHC	0.850	MitoCarta gene
AK3	0.728	MitoCarta gene	COX5A	0.931	MitoCarta gene	GLRX5	0.628	MitoCarta gene	MPC1	0.636	MitoCarta gene
AKR1B10	2.015	MitoCarta gene	COX6A1	0.650	MitoCarta gene	GLUD1	0.926	MitoCarta gene	MPV17L	0.900	MitoCarta gene
ALDH18A1	1.142	MitoCarta gene	COX6A2	0.808	MitoCarta gene	GOLPH3	0.824	MitoCarta gene	MRPL15	0.653	MitoCarta gene
ALDH1L2	1.432	MitoCarta gene	COX6B1	0.686	MitoCarta gene	GPAM	1.145	MitoCarta gene	MRPL19	0.886	MitoCarta gene
ALDH2	0.742	MitoCarta gene	COX7A2	0.617	MitoCarta gene	GPD2	1.127	MitoCarta gene	MRPL3	0.844	MitoCarta gene
ALDH5A1	1.128	MitoCarta gene	COX7A2L	0.608	MitoCarta gene	GPX1	0.968	MitoCarta gene	MRPL32	0.616	MitoCarta gene
ALKBH1	0.626	MitoCarta gene	COX7B2	1.286	MitoCarta gene	GRPEL2	0.847	MitoCarta gene	MRPL33	1.009	MitoCarta gene
ANGEL2	0.888	MitoCarta gene	COX8A	0.869	MitoCarta gene	GRSF1	0.858	MitoCarta gene	MRPL35	0.749	MitoCarta gene
APOO	1.242	MitoCarta gene	CPOX	1.000	MitoCarta gene	GSR	0.890	MitoCarta gene	MRPL38	0.750	MitoCarta gene
ARMC10	0.766	MitoCarta gene	CPT1A	0.919	MitoCarta gene	GTPBP6	0.841	MitoCarta gene	MRPL39	0.829	MitoCarta gene
ARMCX1	0.769	MitoCarta gene	CROT	2.108	MitoCarta gene	GUK1	0.610	MitoCarta gene	MRPL42	0.987	MitoCarta gene
ARMCX2	0.927	MitoCarta gene	CRY1	1.237	MitoCarta gene	HCCS	1.489	MitoCarta gene	MRPL44	1.263	MitoCarta gene
ARMCX3	0.679	MitoCarta gene	CRYZ	1.048	MitoCarta gene	HEBP1	0.983	MitoCarta gene	MRPL45	0.705	MitoCarta gene
ATAD1	0.935	MitoCarta gene	CYB5B	1.143	MitoCarta gene	HIBADH	1.049	MitoCarta gene	MRPL47	0.984	MitoCarta gene
ATP23	0.874	MitoCarta gene	CYB5R3	1.149	MitoCarta gene	HIGD1A	1.385	MitoCarta gene	MRPL48	0.736	MitoCarta gene
ATPAF1	0.801	MitoCarta gene	CYCS	1.008	MitoCarta gene	HINT3	0.642	MitoCarta gene	MRPL50	0.951	MitoCarta gene
BBC3	1.532	MitoCarta gene	CYP11B1	0.829	MitoCarta gene	HSD17B4	0.880	MitoCarta gene	MRPL52	0.728	MitoCarta gene
BCKDHB	0.822	MitoCarta gene	CYP11B2	1.552	MitoCarta gene	HSDL2	0.659	MitoCarta gene	MRPL53	0.658	MitoCarta gene
BCL2L10	0.784	MitoCarta gene	DAP3	0.657	MitoCarta gene	HSPA9	0.873	MitoCarta gene	MRPL57	0.602	MitoCarta gene
BCL2L13	0.767	MitoCarta gene	DARS2	0.712	MitoCarta gene	HSPD1	0.684	MitoCarta gene	MRPL58	0.774	MitoCarta gene
BCL2L2	0.584	MitoCarta gene	DBI	0.980	MitoCarta gene	HTATIP2	1.172	MitoCarta gene	MRPS10	0.940	MitoCarta gene
BID	2.269	MitoCarta gene	DDX28	0.757	MitoCarta gene	IDE	1.095	MitoCarta gene	MRPS12	0.728	MitoCarta gene
BIK	0.583	MitoCarta gene	DECR1	0.724	MitoCarta gene	IDH3A	1.225	MitoCarta gene	MRPS14	0.607	MitoCarta gene
BNIP3	0.682	MitoCarta gene	DLAT	0.912	MitoCarta gene	IFI27	0.914	MitoCarta gene	MRPS17	0.965	MitoCarta gene
BNIP3L	0.923	MitoCarta gene	DLD	1.088	MitoCarta gene	IMMP2L	0.713	MitoCarta gene	MRPS18B	0.889	MitoCarta gene
BOLA3	0.897	MitoCarta gene	DNAJC15	0.614	MitoCarta gene	IMMT	0.875	MitoCarta gene	MRPS18C	0.724	MitoCarta gene
C12orf65	1.253	MitoCarta gene	DNAJC28	0.720	MitoCarta gene	ISCA1	1.169	MitoCarta gene	MRPS22	0.649	MitoCarta gene
C15orf48	2.315	MitoCarta gene	DNM1L	0.727	MitoCarta gene	LACTB	0.871	MitoCarta gene	MRPS24	0.690	MitoCarta gene
C15orf61	0.741	MitoCarta gene	DTYMK	0.833	MitoCarta gene	LAP3	1.090	MitoCarta gene	MRPS25	0.721	MitoCarta gene
C1QBP	1.034	MitoCarta gene	EARS2	0.827	MitoCarta gene	LDHAL6B	1.510	MitoCarta gene	MRPS31	0.603	MitoCarta gene
C2orf69	0.788	MitoCarta gene	ECHDC1	0.782	MitoCarta gene	LDHB	0.672	MitoCarta gene	MRPS34	0.847	MitoCarta gene
C3orf33	1.192	MitoCarta gene	ELAC2	0.636	MitoCarta gene	LETM2	0.774	MitoCarta gene	MRPS36	0.787	MitoCarta gene
CA5A	1.180	MitoCarta gene	ETFA	0.668	MitoCarta gene	LIAS	0.669	MitoCarta gene	MRPS9	0.839	MitoCarta gene
CA5B	1.702	MitoCarta gene	ETFDH	1.054	MitoCarta gene	LIG3	1.182	MitoCarta gene	MSRA	0.594	MitoCarta gene
CASP8	1.982	MitoCarta gene	ETHE1	0.784	MitoCarta gene	LRPPRC	0.665	MitoCarta gene	MTCH2	0.919	MitoCarta gene
CASP9	0.677	MitoCarta gene	EXD2	0.743	MitoCarta gene	LYPLA1	0.948	MitoCarta gene	MTERF1	0.660	MitoCarta gene
CAT	0.801	MitoCarta gene	EXOG	1.380	MitoCarta gene	LYRM2	0.884	MitoCarta gene	MTERF2	0.597	MitoCarta gene

Continue for Supplementary Table 1C. Complete list of 400 mitoCarta genes and 59 ER stress genes upregulated in renal biopsy specimens collected from CKD patients. This gene list is related to Figure 1C.

Gene symbol	log2FC	Category	Gene symbol	log2FC	Category	Gene symbol	log2FC	Category	Gene symbol	log2FC	Category
MTERF3	0.762	MitoCarta gene	PEX11B	0.809	MitoCarta gene	SPTLC2	1.503	MitoCarta gene	BCL2L1	0.946	ER stress and MitoCarta gene
MTERF4	0.800	MitoCarta gene	PIF1	0.807	MitoCarta gene	SSBP1	1.245	MitoCarta gene	BCL2L11	1.239	ER stress and MitoCarta gene
MTFMT	0.852	MitoCarta gene	PLGRKT	0.876	MitoCarta gene	STAR	1.132	MitoCarta gene	CASP3	0.835	ER stress and MitoCarta gene
MTFR2	1.273	MitoCarta gene	PLSCR3	0.894	MitoCarta gene	STOM	1.937	MitoCarta gene	GTF2I	0.780	ER stress
MTHFD1L	1.561	MitoCarta gene	PMAIP1	1.491	MitoCarta gene	STX17	0.614	MitoCarta gene	CREB1	1.756	ER stress
MTHFD2L	0.796	MitoCarta gene	PNPLA8	1.148	MitoCarta gene	SUCLA2	0.803	MitoCarta gene	CREB3L1	0.856	ER stress
MTIF2	0.853	MitoCarta gene	PNPO	0.930	MitoCarta gene	SUPV3L1	0.780	MitoCarta gene	CREBBP	0.723	ER stress
MTO1	0.591	MitoCarta gene	PNPT1	0.847	MitoCarta gene	SYNJ2BP	0.747	MitoCarta gene	DBF4	1.555	ER stress
MTPAP	1.085	MitoCarta gene	POLB	1.437	MitoCarta gene	TAMM41	0.631	MitoCarta gene	DNAJC10	1.106	ER stress
MTRF1	0.708	MitoCarta gene	PPA2	0.726	MitoCarta gene	TCAIM	0.791	MitoCarta gene	DNAJC3	1.423	ER stress
MTRF1L	0.657	MitoCarta gene	PPIF	1.079	MitoCarta gene	TDRKH	1.181	MitoCarta gene	EDEM1	1.194	ER stress
NADK2	0.881	MitoCarta gene	PPM1K	1.554	MitoCarta gene	TEFM	0.608	MitoCarta gene	EIF2A	0.795	ER stress
NARS2	0.596	MitoCarta gene	PPTC7	0.977	MitoCarta gene	TFB2M	0.757	MitoCarta gene	EIF2AK3	0.904	ER stress
NAXD	1.073	MitoCarta gene	PRDX4	0.804	MitoCarta gene	THEM5	0.602	MitoCarta gene	ERN1	0.716	ER stress
NDUFA4	0.650	MitoCarta gene	PRDX6	0.629	MitoCarta gene	THG1L	0.821	MitoCarta gene	ERP44	1.193	ER stress
NDUFA5	0.872	MitoCarta gene	PRELID2	0.701	MitoCarta gene	THNSL1	0.731	MitoCarta gene	CALR	1.263	ER stress
NDUFA7	0.676	MitoCarta gene	PRELID3B	1.090	MitoCarta gene	TIMM13	0.662	MitoCarta gene	CANX	1.129	ER stress
NDUFA8	0.626	MitoCarta gene	PREPL	1.147	MitoCarta gene	TIMM17A	0.848	MitoCarta gene	CASP12	1.394	ER stress
NDUFAF1	0.838	MitoCarta gene	PTCD1	0.915	MitoCarta gene	TIMM21	0.760	MitoCarta gene	CCT2	1.079	ER stress
NDUFAF3	1.168	MitoCarta gene	PTCD2	1.113	MitoCarta gene	TIMM50	0.633	MitoCarta gene	CCT4	0.806	ER stress
NDUFAF4	0.997	MitoCarta gene	PTRH1	0.891	MitoCarta gene	TIMM8A	0.790	MitoCarta gene	CEBPB	0.738	ER stress
NDUFB4	0.823	MitoCarta gene	PTRH2	1.081	MitoCarta gene	TIMM9	0.725	MitoCarta gene	ATF6	1.036	ER stress
NDUFB5	0.697	MitoCarta gene	PXMP4	0.991	MitoCarta gene	TIMMDC1	0.752	MitoCarta gene	ATXN3	1.109	ER stress
NDUFC1	0.769	MitoCarta gene	QRSL1	0.813	MitoCarta gene	TMEM126A	0.699	MitoCarta gene	HERPUD1	0.844	ER stress
NDUFS1	0.790	MitoCarta gene	RARS2	1.335	MitoCarta gene	TMEM143	0.620	MitoCarta gene	HIF1A	1.258	ER stress
NDUFS4	0.612	MitoCarta gene	RDH14	0.995	MitoCarta gene	TMEM205	0.695	MitoCarta gene	HSPA4	1.026	ER stress
NDUFS7	0.693	MitoCarta gene	REXO2	0.815	MitoCarta gene	TMEM65	0.841	MitoCarta gene	HSPA5	1.108	ER stress
NDUFV3	0.655	MitoCarta gene	RFK	1.133	MitoCarta gene	TOMM70	0.869	MitoCarta gene	HTRA4	1.173	ER stress
NFS1	0.969	MitoCarta gene	RHOT1	0.691	MitoCarta gene	TRIT1	0.729	MitoCarta gene	INSIG2	0.938	ER stress
NGRN	1.143	MitoCarta gene	RNASEH1	1.426	MitoCarta gene	TRMT10C	0.935	MitoCarta gene	MANF	1.292	ER stress
NLN	2.062	MitoCarta gene	ROMO1	1.094	MitoCarta gene	TRNT1	1.084	MitoCarta gene	MAP2K4	1.060	ER stress
NNT	0.968	MitoCarta gene	RPIA	0.932	MitoCarta gene	TXNRD1	1.487	MitoCarta gene	MAPK8	0.684	ER stress
NOA1	0.658	MitoCarta gene	SCP2	1.258	MitoCarta gene	UCP1	1.876	MitoCarta gene	MBTPS1	0.751	ER stress
NOCT	1.571	MitoCarta gene	SDHAF3	0.651	MitoCarta gene	MRS2	0.737	MitoCarta gene	MBTPS2	1.481	ER stress
NSUN2	0.675	MitoCarta gene	SDHAF4	0.660	MitoCarta gene	UNG	0.815	MitoCarta gene	NFE2L2	1.081	ER stress
NSUN3	0.630	MitoCarta gene	SERAC1	1.492	MitoCarta gene	UQCR10	0.777	MitoCarta gene	NFKB1	0.766	ER stress
NT5DC3	1.185	MitoCarta gene	SETD9	0.908	MitoCarta gene	UQCR11	0.683	MitoCarta gene	SERP1	0.647	ER stress
NUBPL	0.927	MitoCarta gene	SFXN1	1.190	MitoCarta gene	UQCRC2	0.647	MitoCarta gene	SIL1	0.643	ER stress
NUDT19	1.569	MitoCarta gene	SIRT5	0.751	MitoCarta gene	UQCRH	0.881	MitoCarta gene	PCNA	1.098	ER stress
OCIAD1	0.953	MitoCarta gene	SLC25A21	0.683	MitoCarta gene	UQCRQ	0.634	MitoCarta gene	PDIA3	1.097	ER stress
OCIAD2	0.979	MitoCarta gene	SLC25A22	0.933	MitoCarta gene	VDAC2	0.843	MitoCarta gene	SLC17A2	1.209	ER stress
OMA1	1.106	MitoCarta gene	SLC25A23	0.642	MitoCarta gene	VWA8	1.074	MitoCarta gene	TCP1	0.876	ER stress
OPA1	0.611	MitoCarta gene	SLC25A24	0.842	MitoCarta gene	WARS2	1.025	MitoCarta gene	PPP1CC	1.009	ER stress
OPA3	0.926	MitoCarta gene	SLC25A25	0.720	MitoCarta gene	YARS2	0.836	MitoCarta gene	RPN1	1.147	ER stress
OSBPL1A	1.525	MitoCarta gene	SLC25A26	0.868	MitoCarta gene	YME1L1	1.405	MitoCarta gene	RRM1	1.014	ER stress
OSGEPL1	0.924	MitoCarta gene	SLC25A31	1.634	MitoCarta gene	APOOL	0.899	MitoCarta gene	SEC63	1.310	ER stress
OXNAD1	0.774	MitoCarta gene	SLC25A32	0.997	MitoCarta gene	DBT	1.076	MitoCarta gene	SEL1L	1.683	ER stress
PABPC5	1.518	MitoCarta gene	SLC25A40	1.302	MitoCarta gene	TFAM	1.001	MitoCarta gene	TRIB3	1.349	ER stress
PAICS	0.725	MitoCarta gene	SLC25A41	1.075	MitoCarta gene	NDUFC2	0.990	MitoCarta gene	UBXN4	1.098	ER stress
PANK2	0.790	MitoCarta gene	SLC25A43	1.437	MitoCarta gene	ISCA2	0.750	MitoCarta gene	UHRF1	1.081	ER stress
PARS2	0.663	MitoCarta gene	SLC25A51	1.083	MitoCarta gene	LACTB2	1.239	MitoCarta gene	XBP1	1.206	ER stress
PCCA	0.766	MitoCarta gene	SLC25A52	1.105	MitoCarta gene	NUDT9	0.958	MitoCarta gene	VCP	0.688	ER stress
PDHA2	1.543	MitoCarta gene	SND1	0.633	MitoCarta gene	GLUD2	0.587	MitoCarta gene	VIMP	0.753	ER stress
PDHX	0.877	MitoCarta gene	SOD1	0.674	MitoCarta gene	SOD2	1.137	MitoCarta gene	USP14	0.820	ER stress
PDK3	0.916	MitoCarta gene	SPATA19	1.332	MitoCarta gene	LYRM1	0.854	MitoCarta gene	UGGT1	1.049	ER stress
PDP1	0.999	MitoCarta gene	SPHKAP	1.390	MitoCarta gene	MFN2	0.777	ER stress and MitoCarta gene	DERL1	1.649	ER stress
PDSS1	0.899	MitoCarta gene	SPIRE1	1.018	MitoCarta gene	BCL2	0.827	ER stress and MitoCarta gene	RRM2	1.082	ER stress

Supplementary Table 2. FMO3 expression in different cell types in physiological and pathological conditions

Physiological/ pathological Conditions	FMO3 expressing cells	PMID
COVID-19 lung	Endothelial cells, Fibroblast, Epithelial cells, B cells, Mast cells	33915568
Immune cell from human lung tumor	B cells, T cells, DC, RBC, Plasma cells, NK cells, Neutrophils, Mast cells, Fibroblast, Monocyte/Macrophage	30979687
Normal human heart	Endothelial cells, Fibroblast, Pericytes	32403949
COVID-19 heart	Fibroblast, Cardiomyocytes, vascular Endothelial cells, Pericytes, SMCs	*
Mouse glomerulus diseases (Doxorubicin injury)	Endothelial cells, Mesangial cell	32651223

Supplementary Table 3. ScRNA-Seq data mining * https://singlecell.broadinstitute.org/single_cell/study/SCP1214/ showed the mRNA expression of FMOs family members in physiological and pathological conditions in various cell types in different organs.

Physiological/ Pathological conditions	FMO1 expressing cells	FMO2 expressing cells	FMO4 expressing cells	FMO5 expressing cells	FMO6 expressing cells	PMID
COVID-19 lung	ECs, fibroblast, T cells, and mast cells	ECs, fibroblast, epithelial cells, B cells, mast cells, and myeloid cells	ECs, fibroblast, epithelial cells, B cells, T cells, mast cells, and neuronal cells	ECs, fibroblast, epithelial cells, B cells, T cells, mast cells, myeloid cells, and neuronal cells		33915568
Immune cell from human lung tumor	monocyte/macrophage, B cells, T cells, DCs, and fibroblast	ECs, fibroblast, SMCs, and T cells	ECs, B cells, T cells, DCs, and Monocyte/Macrophage	ECs, B cells, T cells, mast cells monocyte/macrophage, and DC	-	30979687
Normal human heart	ECs, fibroblast, and pericytes	ECs, fibroblast, VSMCs, macrophages, pericytes, cardiomyocytes, and adipocytes	ECs, fibroblast, pericytes, VSMCs, macrophages, cardiomyocytes, and adipocytes	ECs, fibroblast, pericytes, cardiomyocytes, VSMCs adipocytes, macrophages	-	32403949
COVID-19 heart	ECs, fibroblast, pericytes, and cardiomyocytes	ECs, macrophages, SMCs, pericytes, and cardiomyocytes	ECs, macrophages, SMCs, pericytes, and cardiomyocytes	ECs, macrophages, SMCs, pericytes, and cardiomyocytes	-	*
Mouse glomerulus diseases (Doxorubicin injury)	ECs, ,mesangial cells, and podocytes	ECs, mesangial cells, and podocytes	ECs	ECs, mesangial cells, immune cells, podocytes, and SMCs	-	32651223
Aging mouse brain	ECs, astrocytes, arachnoid barrier cells, pericytes, olfactory ensheathing glia, and oligodendrocyte precursor cells	ECs, pericytes, and astrocytes	-	ECs, arachnoid barrier cells, astrocyte-restricted precursors, microglia, pericytes, macrophages, and olfactory ensheathing glia	-	31551601

Supplementary Table 4. TMAO upregulated eight CD membrane proteins and their interaction partners and functions

Gene symbol	Log_2FC	interaction partner	Function	PMID
KLRC1 (CD159a)	3.06	MHC-I molecules	Regulate humoral and cell-mediated immunity	17172651
NCAM1 (CD56)	2.31	FGFR, GDNF, GFL/GFRα co-receptor	Inflammatory response	12837245 15528382
CD6	2.10	CD166, CD318	A costimulatory molecule that synergizes with the TCR to enhance/inhibit T cell activation	26146185
CD248	1.90	Fibronectin metastasis-related protein Mac-2 BP/90K	Vascular inflammation and angiogenesis	30847027 33737161
MUC1	1.12	SELE/ICAM1	Vascular inflammation and angiogenesis	21927028
DPP4 (CD26)	0.76	Caveolin-1, fibronectin, ADA, and CXCR4	Glucose metabolism, regulation of immune system, modulating T cell activation and signal transduction	32061923
IL6R (CD126)	0.73	IL-6 and IL-1	Pro-inflammatory properties	24986424
SELE (CD62E)	0.72	ESL-1, PSGL-1, CD44, DR3, LAMP1	Cellular adhesion and vascular inflammation	29403469

Table 5. Subcellular locations of the 12 TMAO activated kinases from protein subcellular location database COMPARTMENTS and experimentally verified protein database Human Protein Atlas (HPA)

kinases	Subcellular locations from COMPARTMENTS	Subcellular locations from the Human Protein Atlas (HPA)
P38 alpha (MAPK14)	Extracellular, nucleus, cytosol	Intracellular
ERK1/2 (MAPK3/1)	Plasma membrane, extracellular, cytoskeleton, mitochondrion, nucleus, endosome, cytosol, golgi apparatus, endoplasmic reticulum	NA
JNK1/2/3 (MAPK8/9/10)	Nucleus, cytosol	
GSK3A/B	Mitochondrion, nucleus, cytosol, plasma membrane, cytoskeleton, nucleus	Intracellular, Membrane
EGFR	Plasma membrane, extracellular, nucleus, endosome	Intracellular, Membrane, Secreted
MSK1/2 (RPS6KA5/4)	Nucleus, cytosol	Intracellular
AMPK alpha1 (PRKAA1)	Nucleus, cytosol	Intracellular
AKT1/2/3	Plasma membrane, cytoskeleton, nucleus, cytosol	Intracellular
MTOR	Nucleus, cytosol, Lysosome	Intracellular
CREB1	Nucleus, mitochondrion	Intracellular
AMPK alpha2 (PRKAA2)	Nucleus, cytosol,	Membrane
PDGFRB	Plasma membrane, nucleus, lysosome	Intracellular, Membrane

Supplementary Table 6. The Table showed that TNF-α increased ICAM-1 expression and that TNFα receptor knockdown reduced ICAM-1 expression in endothelial cells, indicating that TNF-α upregulation is associated with the upregulation of ICAM-1.

Experimental condition	Effects on ICAM1	Cell type	Pathway	PMID
TNF-α	Induced ICAM-1 expression	ECs	Tyrosine kinase pathway	8937718
TNF-α	Induced ICAM-1 expression	Epithelial cells	PC-PLC/DAG/PKC pathway	10837365
TNF-α	Induced ICAM-1 expression and ROS generation	ECs	IKKβ/IκBα-mediated activation of NF-κB p65	29021525
TNF-α	Induced ICAM-1 expression	Vascular SMCs		8095156
TNF-α	Induced ICAM-1 expression	ECs	Glutathione peroxidase (GPx)	9607608
TNFR2$^{-/-}$	Reduced ICAM1 expression	ECs		17068152
TNFR1, 2 $^{-/-}$	Reduced ICAM1 expression	ECs		23056240
oxLDL training	Induced ICAM-1 expression	HAECs	HIF1α-dependent manner	32726647

Supplementary Table 7. TMAO-activated kinases play a critical role in glycolysis and endothelial cell function

**Kinases**	**Endothelial cell activation**	PMID	**Glycolysis**	**PMID**
P38	Contributes to endothelial cell activation/dysfunction	25746230<br>12576315	Enhances Glycolysis	25925563
ERK1/2	Mediates endothelial cell dysfunction	32863203	Promotes aerobic glycolytic	30487597
JNK1/2/3	Mediated TNF-a induced ICAM expression and EC activation	15979056	Promotes glycolysis through activation of PFK-1	18469002
EGFR	Contributes to EC dysfunction and oxidative stress	31892280<br>32548701	Enhances Aerobic Glycolysis	26759242<br>24928511
GSK3	Contributes to endothelial dysfunction	21549192	GSK-3β inhibition leads to down-regulation of glycolysis-related key enzymes	31404613
AMPK alpha1			Mediates increase in glycolysis	22001850
AMPK alpha2				28974774<br>26322680
AKT1/2/3	Increased EC activation and vascular EC damage	29956071	Increases glycolysis	31068941<br>32051533
TOR	Increased VCAM1 expression and EC activation	30462552	Activates glycolysis	26023239
CREB	Contributes to endothelial dysfunction	21549192<br>1476188	Increases glycolysis	29228623
PDGFR-β			Promotes aerobic glycolysis	34678088

Supplementary Table 8. Human primers used for real-time PCR

Human primers	F (5’-3’)	R (5’-3’)
Human ICAM1	TCTACGCTGACAATGAATCCTG	AGGGCCACTCAAATGAATCTC
Human ACTB	ACCTTCTACAATGAGCTGCG	CCTGGATAGCAACGTACATG
Human TNFα	ACTTTGGAGTGATCGGCC	GCTTGAGGGTTTGCTACAAC
Human IL1β	ATGCACCTGTACGATCACTG	ACAAAGGACATGGAGAACACC
Human SELE	AAGTTCGCCTGTCCTGAAG	CAGAAAGTCCAGCTACCAAGG
Human ACE	TGGTGTGGAACGAGTATGC	AGGGTGTGGTTGGCTATTTG
Human PLAT	AAACCCAGATCGAGACTCAAAG	ACCCATTCCCAAAGTAGCAG
